# Clinical course and prognosis of chronic autoimmune neuropathies requiring intensive care: a retrospective cohort study

**DOI:** 10.1007/s00415-026-13694-4

**Published:** 2026-02-25

**Authors:** Hannah Preßler, Lisa Schwarz, Simon Streit, Annette Aigner, Alicia Schleicher, Frauke Stascheit, Friederike A. Arlt, Viktoria Zinnow, Tatjana Khorassani, Harald Prüss, Wolfgang Böhmerle, Andreas Meisel, Werner Stenzel, Franziska Scheibe

**Affiliations:** 1 Department of Neurology With Experimental Neurology, Charité-Universitätsmedizin Berlin, Corporate Member of Freie Universität Berlin, Humboldt-Universität Berlin, Berlin, Germany; 2 German Center for Neurodegenerative Diseases (DZNE) Berlin, Berlin, Germany; 3 Neuroscience Clinical Research Center, Charité – Universitätsmedizin Berlin, corporate member of Freie Universität Berlin and Humboldt Universität zu Berlin, Berlin, Germany; 4 Department of Neuropathology, Charité-Universitätsmedizin Berlin, Corporate Member of Freie Universität Berlin, Humboldt-Universität Berlin, Berlin, Germany; 5 Institute of Biometry and Clinical Epidemiology, Charité-Universitätsmedizin Berlin, Corporate Member of Freie Universität Berlin, Humboldt-Universität Berlin, Berlin, Germany; 6 Department of Laboratory Medicine and Pathology, Mayo Clinic, Rochester, USA; 7 Center for Stroke Research Berlin, Charité-Universitätsmedizin Berlin, Corporate Member of Freie Universität Berlin, Humboldt-Universität Berlin, Berlin, Germany; 8 Department of Neurology, Asklepios Klinik Hamburg Harburg, Hamburg, Germany

**Keywords:** Chronic autoimmune neuropathies, Intensive care unit, Daratumumab, Autologous stem cell transplantation

## Abstract

**Background and objective:**

Data on chronic autoimmune neuropathies (CAN) requiring intensive care unit (ICU) treatment are limited. This study investigated clinical and neuropathological features, immunotherapies, and outcomes of ICU-treated CAN patients.

**Methods:**

Retrospectively, we analyzed patients with CAN admitted to the ICU between 2007 and 2024. Outcomes, assessed by Inflammatory Neuropathy Cause and Treatment (INCAT) score and modified Rankin scale (mRS), were compared to age, sex, and diagnosis-matched non-ICU outpatients using ordinal and binary logistic regression.

**Results:**

Among 21 included patients (chronic inflammatory demyelinating neuropathies (CIDP): *n* = 15, other CAN: *n* = 6), 95% required mechanical ventilation and exhibited severe tetraparesis at disease nadir. Biopsies from CIDP patients revealed moderate-to-severe chronic axonal loss with variable CD8 + T-cell infiltration (9/11), and complement deposition (C5b-9) was detected in all samples (*n* = 8/8). All patients received first-line immunotherapy at ICU, 62% required “escalation” (rituximab: *n* = 13, cyclophosphamide: *n* = 3). Five (24%) remained refractory, receiving daratumumab (*n* = 3), efgartigimod (*n* = 1), or autologous stem cell transplantation (*n* = 1). Six patients (29%) died, whereas survivors showed marked improvement with median change of − 2 mRS points (95% CI − 5 to − 2) and − 6 INCAT points (95% CI − 8 to− 5) at last follow-up. However, ICU-treated patients had significantly higher odds of worse long-term outcomes than matched non-ICU patients (adjusted cumulative OR: mRS 7.1 95% CI 1.9–27.3, INCAT 6.4 95% CI 1.7–23.2).

**Conclusion:**

Severe CAN requiring ICU treatment is associated with high mortality, but meaningful recovery is possible in survivors. Neuropathology confirmed combined cellular and humoral mechanisms, supporting personalized, mechanism-guided therapeutic approaches.

**Supplementary Information:**

The online version contains supplementary material available at 10.1007/s00415-026-13694-4.

## Introduction

Chronic autoimmune neuropathies (CANs) are immune-mediated disorders of the peripheral nervous system with variable severity and unpredictable disease courses [1]. Common subtypes include chronic inflammatory demyelinating polyradiculoneuropathy (CIDP), multifocal motor neuropathy (MMN), and immunoglobulin M (IgM)-associated neuropathies, with or without antibodies (Abs) against myelin-associated glycoprotein (MAG) [2].

More recently, autoimmune para- and nodopathies (AN) have been recognized as distinct entities in the current EAN/PNS guidelines, showing poor response to first-line CIDP therapies but often favorable response to B-cell depletion treatment [3–5]. Unlike the acute onset seen in AN, CAN mostly follows a more slowly progressive or relapsing disease course [3]. Plasma exchange (PLEX) is frequently considered during acute deterioration, particularly in patients with insufficient responses to intravenous immunoglobulins (IVIG) or corticosteroids [6, 7]; however up to 25% of CAN patients do not respond to first-line treatments, and about 15% remain resistant to all established immunotherapies [7], underscoring the need for novel therapeutic approaches. Case reports and case series suggest benefits of rituximab, cyclophosphamide, and other (chemo)-therapeutic agents [7–9], yet standardized “escalation” strategies are lacking [3]. While intensive care unit (ICU) management of acute neuropathies such as Guillain–Barré syndrome (GBS) is well established [10, 11], the frequency, treatment strategy, and prognosis of CAN crises requiring intensive care remain poorly characterized [12, 13].

We therefore analyzed ICU-treated patients with CAN, including clinical, electrophysiological, and neuropathological assessments, along with treatment approaches and outcomes. Additionally, we compared therapies and outcomes to non-ICU CAN outpatients from our clinic and pooled data of ICU-treated CAN patients derived from published literature for comparison.

## Methods

### Study design, patient selection, and data collection

This single-center retrospective study included patients diagnosed with CAN and treated at the neurological ICU at Charité–Universitätsmedizin Berlin, Germany, between May 2007 and November 2024. For comparison, we included patients with similar diagnoses presenting to the outpatient clinic for inflammatory neuropathies between January and December 2024. Additionally, articles published between March 1987 and February 2025 were used to pool data reported on other ICU-treated CAN patients.

### ICU-treated cohort

For definition of clinical entities, we adhered to the relevant guidelines and previous studies, e.g., CIDP and its variants [3, 14], ANs [5], anti-MAG neuropathy [15], and MMN [16]. Owing to the long study period and changing guidelines regarding CIDP and its variants over the years, we reassessed the initial clinical diagnoses in accordance with the current European Federation of Neurological Societies/Peripheral Nerve Society guideline (EAN/PNS) [3] and retrospectively applied these criteria to the clinical and diagnostic information available at the time of the initial assessment, as well as later stages during the disease course. Consequently, two patients who had initially been diagnosed with CIDP were retrospectively reclassified as AN, following the introduction of diagnostic assays for paranodal and nodal antibodies, while the remaining patients continued to fulfill the updated diagnostic criteria. Patients were diagnosed with chronic axonal autoimmune polyneuropathy (CIAP) when all three of the following criteria were met: (1) acquired, chronic progressive, or relapsing polyneuropathy (symmetrical or asymmetrical) with progression lasting > 2 months, (2) electrophysiological evidence of axonal neuropathy in at least two nerves without demyelinating features, (3) clear and clinically meaningful response to immunotherapy [17]. Acute-onset CAN was diagnosed in patients who showed renewed deterioration beyond 8 weeks from onset or experienced more than three deteriorations during medication tapering [3, 18]. Data on demography, clinical symptoms, and applied diagnostics (laboratory tests, CSF findings, imaging, electrophysiology) were extracted from the hospital information system and medical records.

### Non-ICU cohort

Patients presenting to the outpatient clinic for inflammatory neuropathies at Charité–Universitätsmedizin Berlin were screened for inclusion based on diagnoses comparable to those of the ICU-treated CAN cohort. Patients who had already received intensive care treatment for CAN—either at our institution or at an external center—prior to their outpatient visit were excluded from comparative analyses with the ICU cohort.

### Literature-derived ICU cohort

Publications containing individual-level data on ICU-treated CAN patients were identified by a literature review following the Preferred Reporting Items for Systematic Review and Meta-Analyses (PRISMA) guidelines (Supplementary Fig. S1), and the reported information pooled. Details on the systematic search strategy and inclusion criteria are provided in the Supplementary Methods.

### Laboratory

Serum samples were screened for the presence of Abs, including anti-nodal and paranodal Abs (contactin-1, contactin1/contactin-associated protein1 complex, neurofascin-155, neurofacin-186, contactin-associated protein1), anti-ganglioside IgG/IgM Abs (GM1, GM2, GM3, GD1a, GD1b, GT1b, GQ1b), and anti-MAG Abs using line blots, ELISA, and cell-based assays (Labor Berlin GmbH, Berlin Germany, Euroimmun Labor, Lübeck Germany). Serum neurofilament light chain (sNfL) concentrations were quantified using the Simoa® NF-Light assay on an SR-S analyzer (Quanterix, Boston, MA, USA), following the manufacturer’s instructions [19]. Age-adjusted z-scores were generated for sNfL concentrations [20].

### Histopathology

Histopathological assessments were performed in ICU-treated patients who had undergone sural nerve biopsy as part of the diagnostic workup and did not opt out of biomaterial use. As the degree of chronic nerve damage (e.g., axonal loss, remyelination markers) depends on time since disease onset, biopsy timing was categorized into acute (< 30 days), subacute (30–90 days), or chronic (> 90 days after onset), reflecting disease progression. As chronic nerve damage is additionally influenced by the time since ICU admission, this time interval was also recorded. Together, these predefined intervals account for the expected increase in chronic pathological changes with longer disease duration and ICU stay, respectively (provided in Table 5).

Slides prepared during the initial diagnostic workup were used for analysis; when required, missing sections or stains were completed according to standard laboratory protocols. We assessed five histological features: (1) myelinated fiber density as an indicator of chronic axonal damage, (2) number of CD8 + T cells as an indicator of endoneurial lymphocytic infiltration, (3) presence of thinly myelinated fibers as an indicator of de- and remyelination and regenerative clusters as indicator of persistent axonal damage, (4) presence of acutely degenerating axons as a sign of ongoing nerve injury, and (5) presence of complement deposits on endoneural capillaries.

The density of myelinated fibers was assessed in transverse semithin Sects. (1 µm) fixated in 2.5% glutaraldehyde and stained with methylene blue. Analysis focused on the most artifact-free transverse section in a series of consecutive sections. Sections were digitized using a digital pathology scanner (Aperio GT 450 DX; Leica Biosystems; Wetzlar, Germany) and analyzed using a supervised learning-based morphometry approach validated in our laboratory [21]. Automated detection of fascicles and fibers was manually reviewed, with inaccurately detected fascicles excluded, and results were cross-checked with a semiquantitative assessment ranking fiber density on a scale from 1 (mild chronic axonal damage) to 3 (severe chronic axonal damage). Based on the number of myelinated fibers detected in the automated analysis and our semiquantitative assessments, we categorized patients into three groups: mild damage (> 5000 fibers/µm^2^), moderate damage (2000–5000/µm^2^), and severe damage (< 2000 fibers/µm^2^).

Inflammatory cell infiltration was assessed by quantifying the number of CD8 + cells in cryosections stained for CD8 (Clone UCHL1, M0701, Dako, Glostrup, Denmark). For quantification, we counted CD8 + cells in ten randomly selected high-power fields (HPFs) of 2.4 mm^2^ for each patient sample. We then classified inflammatory infiltration according to the CD8 + cell count as high (> 20 CD8 + cells/10 HPFs), medium (10–20 CD8 + cells/10 HPFs), or low (< 10 CD8 + cells/10 HPFs).

De and remyelination were assessed in semithin sections based on the presence of hypomyelinated fibers and regenerative clusters; ongoing nerve injury was assessed based on the presence of axons with morphological signs of acute degeneration. Two experienced neuropathologists independently rated sections for de and remyelination/acute nerve injury on a scale from 0 (no signs) to 3 (severe). Joint probability of agreement was 46% for each parameter. Following the initial scoring, samples with divergent results were jointly reviewed and consensus was reached through discussion.

Complement deposits on endoneural capillaries were assessed on cryosections stained for C5b-9 (Clone aE11, M777, Dako, Glostrup, Denmark). For semiquantitative assessment, longitudinal and transverse sections were taken into consideration and cases rated for complement deposits on endoneurial capillaries on a scale from 0 (none) to 3 + (prominent).

### Electrophysiology

Nerve conduction studies (NCS) were performed during ICU stay on the median, ulnar, peroneal, tibial (each motor and sensory), and sural (sensory) nerves. Needle electromyographic examinations (EMG) of the tibialis anterior muscle were performed in 20 of 21 patients. Pathological spontaneous activity (PSA) was defined as fibrillations and positive sharp waves in > 10% of needle positions [22]. PSA severity was graded on a semiquantitative scale (1–10), corresponding to the number of PSA-positive needle positions out of ten examined positions within the tibialis anterior muscle: scores 1–3 (10–30%) indicated mild, 4–6 (40–60%) moderate, and 7–10 (70–100%) severe involvement. Demyelination was assessed according to EAN criteria for definite/possible CIDP [3]. Axonal damage was quantified by relative reduction in the amplitudes of the distal compound muscle action potential (CMAP) and sensory nerve action potential (SNAP) using age-adjusted reference values of our medical device (Keypoint G4, Natus, Planegg, Germany). Overall axonal damage was determined based on the number of nerves with reduced CMAP (electroneurography (ENG) score, ranging from 0 (no damage in any limb) to 4 (very severe sensorimotor axonal damage in upper and lower limbs)) with reference values from Stöhr [23] as previously described [22].

### Treatment

Immunotherapies were categorized into four groups: *first-line therapies* (IVIG, subcutaneous immunoglobulins (SCIG), steroids, and PLEX/immunoadsorption (IA)), *other immunotherapies* (e.g., azathioprine, mycophenolate mofetil, cyclosporine, methotrexate), “*escalation” therapies* (e.g., cyclophosphamide, rituximab) applied either sequentially or simultaneously, and *intensified “escalation”* therapies (e.g., efgartigimod, daratumumab, or autologous stem cell transplantation). Efgartigimod was classified as intensified “escalation” therapy, since it was applied before approval as on-label treatment in June 2025 [24].

### Outcome assessment

Outcome scores were retrospectively collected at ICU admission, disease nadir, ICU discharge, and last follow-up. Disability was assessed using the inflammatory neuropathy cause and treatment (INCAT) score (0–10) and modified Rankin scale (mRS, 0–6) with 0 indicating no disability, and 10 or 6 indicating most severe disability (INCAT) or death (mRS), respectively. Common ICU scores included Simplified Acute Physiology Score (SAPS II), Acute Physiology and Chronic Health Classification System II (APACHE2), Sepsis-Related Organ Failure Assessment (SOFA), and Simplified Therapeutic Intervention Scoring System 28 (TISS-28) [25–28]. Follow-up information was obtained from outpatient records and/or readmission/discharge letters (*n* = 13), telephone interviews (*n* = 2), or by official confirmation of death from state authorities (*n* = 6).

For non-ICU patients, only INCAT and mRS at the last clinical visit in 2024 were recorded. Outcomes of the literature-derived ICU cohort were categorized as complete or partial recovery, poor outcome, or death based on the clinical status at the last reported follow-up.

### Statistical analysis

Descriptive results are presented as absolute and relative frequencies for categorical, median, and interquartile range (IQR) for continuous variables, and additionally minimum and maximum where relevant. Intraindividual changes in mRS and INCAT over time were quantified using the median along with a 95% bootstrap confidence interval (CI). Based on the outpatients from the clinic for inflammatory neuropathies with diagnosis spectrum comparable to our ICU cohort, the rate of at least one CAN-related ICU admission from disease onset to last follow-up per 1000 patient-years was calculated, along with a 95% CI For a detailed visualization of treatments applied to each ICU-treated patient over the disease course, a swimmer plot was generated using Python (version 3.9) and additional Python packages [29]. Spearman’s rank correlation coefficients with a 95% bootstrap CIs were used to assess associations between histopathological findings (nerve damage, inflammatory infiltration), clinical outcomes (mRS, INCAT), and axonal damage (ENG) at disease nadir and last follow-up. To assess the effect of time from therapy start to escalation on short and long-term outcome, binary and ordinal logistic regression models were used, additionally adjusted for sex, age at study, acute onset, and tumor to control for potential confounding. To assess the effect of time from disease onset to ICU admission on long-term outcome, we used the same models but adjusted for sex and age at onset only.

To assess the differences between ICU and non-ICU-treated patients, we performed propensity-score matching by age at study inclusion, sex, and diagnosis (CIDP, AN, CIAP, MMN, anti-MAG neuropathy), using a nearest-neighbor algorithm without replacement. A caliper of 0.2 standard deviation of the logit was used [30] and matching quality was visually assessed using standardized mean differences (Supplemental Fig. S3). Based on these parameters, we used a variable matching ratio of 1:10, resulting in best balance in matched variables. The same plots were used to illustrate and assess remaining differences between ICU und non-ICU patients (Supplemental Fig. S4). Due to limited overlap in several variables between the groups, after matching we used adjustment to control for age at onset, steroid or IVIG use, plasmapheresis, escalation therapy, acute onset, and time since onset and infection prior to ICU admission in regression models. Exploratory binary logistic regression was applied for dichotomized outcomes (mRS > 2, INCAT > 3 or death) and ordinal logistic regression was used for ordinal outcomes (mRS, INCAT with death coded as highest category as specified). To rule out that differences in outcomes are due to different follow-up intervals, in a sensitivity analysis we adjusted for time from onset to last follow-up. We used weighted ordinal and binary logistic regression with robust standard errors accounting for the matching, reporting cumulative odds ratios (cOR) or odds ratios (OR), respectively, along with 95% CI. All analyses were performed with R [31] (version 4.3.1) and additional R packages [32–35].

## Results

We identified 21 ICU-treated patients with a median age of 68 years (range: 27–83) at onset and a median disease duration of 3 months (range 0–145). The majority of patient were male (57%, *n* = 12) (Supplementary Table S1). Most were diagnosed with definite or possible CIDP or CIDP variants (*n* = 15/21), including 2 with MGUS IgG kappa and 1 with MADSAM. Other diagnoses included MMN (with MGUS IgM lambda), anti-MAG neuropathy (each *n* = 1/21), CIAP (*n* = 2/21) and AN (*n* = 2/21; anti-NF155-AN [36], anti-NF155/186-AN with MGUS IgG Kappa [37] (each *n* = 1)).

Of 350 patients who presented at the outpatient clinic in 2024, 281 had diagnoses within the same spectrum as the ICU-treated CAN patients. Among these, 12 (4%) reported a CAN-related ICU admission within 2024, 25 patients reported at least one CAN-related ICU admission since disease onset, resulting in an ICU admission rate of 8.8% (95% CI 5.7–12.9) per 1000 patient-years.

### Clinical characteristics of ICU-treated patients

Out of the 21 patients, 10 (47.6%) presented with acute onset (CIDP: *n* = 7, AN: *n* = 2, MADSAM: *n* = 1), all with a preceding viral or bacterial infection 2–6 weeks earlier (Table 1 and Supplementary Table S1). These included upper respiratory tract infection/pneumonia with unknown pathogen (*n* = 4), postoperative *Staphylococcus haemolyticus* sepsis (*n* = 2), *Clostridium* enterocolitis (*n* = 2), SARS-CoV-2 infection (*n* = 2), CMV associated myocarditis (*n* = 1), and a probable flu-like infection with fever up to 39 °C (*n* = 1). Another patient with CIAP and subacute onset reported gastrointestinal infection with unknown pathogen 4 weeks prior to onset. Notably, seven of the ten acute-onset patients (CIDP: *n* = 6, AN: *n* = 1) required ICU admission at first manifestation. Overall, 12 patients (57%) were admitted within the first year after symptom onset, while 9 patients (43%) were admitted after a median disease duration of 4 years.

**Table 1:** Baseline characteristics of matched ICU and non-ICU-treated patients

	Non-ICU cohort		ICU cohort		Total
	All patients	CIDP subgroup	All patients	CIDP subgroup	
*N*°	186	149	20	15	206
Age at symptom onset, median (range)	56.5 (14–81)	56 (14–79)	68.5 (27–83)	71 (27–83)	58 (14–83)
Male sex, *N* (%)	104 (56)	84 (56.4)	11 (55)	7 (46.7)	115 (56)
Onset to therapy, months, Median (range)	22 (0–302–5)	18 (0–302.5)	1 (−6–129)	1 (0–37)	16.5 (3.9–56.9)
Acute onset of symptoms (≤ 2 months), *N* (%)	17 (9.1)	14 (9.4)	9 (45)	8 (53.3)	26 (12.6)
Suspected triggers, *N* (%)					
None/unknown	164 (88.2)	133 (89.3)	8 (40)	5 (33.3)	172 (83.5)
Infection	14 (7.5)	11 (7.4)	10 (50)	8 (53.3)	24 (11.7)
Vaccination	2 (1.1)	1 (0.7)	1 (5)	1 (6.7)	3 (1.5)
Medical procedures	6 (3.2)	4 (2.7)	1 (5)	1 (6.7)	7 (3.4)
Treatment^a^, *N* (%)					
First-line therapy					
Steroids	95 (51.6)	73 (49)	12 (60)	8 (53.3)	107 (51.9)
IVIG/SCIG	160 (86.5)	133 (89.3)	19 (95)	14 (93.3)	179 (86.9)
PLEX/IA^b^	31 (16.8)	27 (18.1)	16 (80)	13 (86.7)	47 (22.8)
First-line therapy before ICU admission					
Only steroids			2 (10)	2 (13.3)	V
Only IVIG			3 (15)	3 (20)	3 (15)
Steroids + IVIG			7 (35)	4 (26.7)	7 (35)
Steroids, IVIG + PLEX			1 (5)	0 (0)	1 (5)
First-line therapy in ICU setting					
Only IVIG			3 (15)	2 (13.3)	3 (15)
Steroids + IVIG			2 (10)	0 (0)	2 (10)
Steroids + IVIG + PLEX			1 (5)	1 (6.7)	1 (5)
Steroids + PLEX			2 (10)	2 (13.3)	2 (10)
IVIG + PLEX			12 (60)	10 (66.7)	12 (60)
“Escalation” therapy, *N* (%)					
Long-term immunosuppressants	19 (10.2)	17 (11.4)	2 (10)	(0)	21 (10.2)
Rituximab	13 (7.0)	10 (6.7)	12 (60)	8 (53.3)	25 (12.1)
Cyclophosphamide	4 (2.2)	2 (1.3)	3 (15)	0 (0)	7 (3)
“Intensified escalation” therapy, *N* (%)					
Daratumumab	0 (0)	0 (0)	2 (10)	2 (13.3)	2 (10)
Efgartigimod	0 (0)	0 (0)	1 (5)	1 (6.7)	1 (5)
Autologous stem cell transplantation	0 (0)	0 (0)	1 (5)	0 (0)	1 (5)
Time from first-line therapy to “escalation” therapy, in months, Median (range)			18 (2.5–124.6)	17.3 (2.5–115.2)	18 (2.5–124.6)
Time from first-line therapy to “intensified escalation” months, Median (range)			46.7 (29.4–117.2)	62.7 (30.8–117–2)	46.7 (29.4–117.2)

Data are presented as median (range) or frequency (%) as appropriate

^a^Multiple selection possible

^b^One ICU-patient needed plasma exchange for clinical stabilization every 1–2 weeks over several months

*CAN* chronic autoimmune neuropathy, *CIDP* chronic inflammatory demyelinating polyneuropathy, *IA* immunoadsorption, *long*-*term IST* long-term immunosuppressants, *IVIG* intravenous immunoglobulin, *PLEX* plasma exchange, *SCIG subcutaneous immunoglobulin *

At ICU admission, nearly all patients presented with sensorimotor weakness (*n* = 20/21). Bulbar or respiratory involvement was observed in 33% (*n* = 7), with one patient with CIDP showing isolated bulbar symptoms (Table 2). ICU admission was primarily due to respiratory insufficiency (*n* = 20, 95%), requiring mechanical ventilation in most cases (95%, mostly invasive) and tracheostomy in 75% (*n* = 16). The median duration of mechanical ventilation was 35 days (range: 10–509, Table 2 and 3). Most patients exhibited tetraparesis with reduced reflexes (*n* = 20, 95%), and cranial nerve involvement (oculomotor disorder, facial or bulbar palsy) was present in 42% (*n* = 9, Supplementary Table S2). Consistent with severe clinical presentation, functional scores at nadir indicated profound disability (median INCAT 10, range 9–10; mRS 5, range 5–5). Most CAN ICU-treated patients presented with comorbidities with cardiovascular disease being most frequent (48%, Supplementary Table S3).

**Table 2:** Demographic and clinical characteristics of patients with severe CAN at ICU

Variable	
Ethnicity, *n* (%)	
Caucasian	19 (90)
Asian	2 (10)
Course of disease at the time point of diagnosis, *n* (%)	
> 8 weeks	11 (52)
≤ 8 weeks (acute-onset CIDP)	10 (48)
Initial symptoms, *n* (%)	
Generalized with bulbar/respiratory involvement	7 (33)
Generalized without bulbar/respiratory involvement	13 (62)
Predominantly bulbar/respiratory involvement	1 (5)
Onset to first hospital admission, median (range) in months	0 (0–127)
Onset to ICU admission, median (range) in months	4.3 (0–146)
Number of hospital stays^a,b^ (disease-related), median (min/max, IQR)	7 (1/20, 1–13)
Duration of ICU stay, median (min/max, IQR) in days, median (min/max, IQR) in days (*n* = 21)	43 (12/309, 31–91)
Indication for ICU admission, *n* (%)	
Respiratory insufficiency	20 (95)
Acute exacerbation^c,d^	1 (5)
Time of follow-up, median (range) in months	19 (0–157)
Patients deceased at last follow-up^e^ (n, % of n = 21)	6 (29)
Age at time point of death in years, median (min/max, IQR)	73 (60/84, 69–77)
Time between last ICU discharge and time point of death, median (min/max, IQR) in days	216 (22/439, 22–554)
Severe complication of the deceased at ICU^f^	5 (83)
Comorbidities of the deceased^g^	6 (100)

^a^ICU stays excluded

^b^two patients were only treated at ICU (no hospital stay at general ward)

^c^disease-related hospital stays were defined as admissions directly attributable to CIDP worsening or acute deterioration; hospitalizations for secondary complications (e.g., falls, trauma, unrelated medical conditions) were not included

^d^in patients with known chronic immune-mediated neuropathy, worsening of motor or sensory deficits without respiratory symptoms

^e^exact causes of deaths remain unknown; however, the presence of severe comorbidities and complications suggest that mortality might not primarily be related to underlying CAN/AN

^f^Complications: severe sepsis (*n* = 4), severe bleeding complication (*n* = 1), ischemic MCA infarction during ICU stay (*n* = 1)

^g^Comorbidities: tumor disease (*n* = 4; rectal carcinoma *n* = 2, rectal and bronchial carcinoma *n* = 1, urothelial carcinoma *n* = 1), IgG4-associated pseudotumor of the medulla oblongata *n* = 1, multimorbidity with previous long-term ICU-stay due to severe pneumogenic sepsis with multiorgan failure

*ICU* intensive care unit, *IQR* interquartile range

**Table 3:** Intensive care treatment and management of patients with CAN

Variable	*N* (%) of *n* = 21
Mechanical ventilation, *n* (% of all patients, *n* = 21)	20 (95)
Invasive	19 (90)
Tracheostomy^a^	16 (76)
Non-Invasive	1 (5)
Duration of mechanical ventilation, median (min/max, IQR), days, *n* = 19	35 (10/509, 21–68)
Duration of respiratory weaning, median (min/max, IQR), days, *n* = 9	8 (4/134, 6–22)
Mechanical ventilation at time of ICU discharge^b^, *n* (%)	12 (57)
Decannulation^c^, *n* (%)	9 (43)
Mechanical ventilation at last follow-up, *n* (%)	2 (10)
Non-invasive	1 (5)
Invasive	1 (5)
Complications^d^, *n* (%)	9 (43)
Nutrition, *n* (%)	
Oral	1 (5)
Parenteral	1 (5)
Tube feeding	8 (38)
PEG	11 (52)
Catecholamine therapy, *n* (%)	21 (100)
Duration of catecholamine therapy, median (min/max, IQR), days, *n* = 20	12 (3/49, 6–20)
Antibiotic therapy, *n* (%)	21 (100)
Duration of antibiotic therapy, median (min/max, IQR), days, *n* = 21	20 (3/187, 12–28)

^a^One patient with severe dysphagia was tracheostomized due to the high risk of aspiration and not because of respiratory failure

^b^Mechanical ventilation at ICU discharge: *n* = 11 via tracheostomy, *n* = 1 on long-term NIV after failed extubation by request

^c^Deceased patients with missing information regarding whether decannulation was performed. *n* = 6, patients with long-term mechanical ventilation *n* = 2

^d^ventilation-associated pneumonia

*PEG* percutaneous endoscopic gastrostomy

Common ICU complications were mainly infectious, occurring in up to 86% of patients (Supplementary Table S4).

### Paraclinical findings in ICU-treated patients

Notably, Ab testing for (para)nodal antigens was performed only in 12 of 21 patients (57%); in 9 patients Ab testing was not done because their ICU admission occurred before commercial assays became available, and follow-up sampling could not be obtained due to early death (*n* = 4) or lack of return to our center (*n* = 5).

NCS during ICU stay revealed severe axonal damage in CAN, with CMAP amplitudes reduced by 89–100% and SNAP amplitudes by 84–100% across all tested nerves, and a median ENG score of 4 (range 0–4). In five patients, at least one nerve demonstrated a partial conduction block. In one patient, an initial partial block progressed to a definite conduction block during follow-up, another developed a new partial block, and in three patients previously observed partial blocks were no longer detectable. PSA was detected in 17 of 19 patients (89%) with available EMG and was graded as mild in 7 (41%), moderate in 8 (47%), and severe in 2 (12%) cases (Supplementary Table S5). In 11 of 21 patients, the same NCS protocol was repeated at follow-up, and the EMG was reassessed in 7 of 21 patients. Five patients (1 during ICU stay, 4 at follow-up) were excluded from ENG scoring due to mainly upper limb involvement, which is not assessable using this scoring system. Follow-up ENG (*n* = 11) showed CMAP increased amplitudes of 54–71% and SNAP amplitudes of 39–53% in upper limbs, compared to minimal gains in lower limbs (CMAP 1–3%, SNAPs by 3%) (Supplementary Table S5). ENG scores improved from a median of 4 (range: 3–4) to 3 (range: 0–4), showing axonal recovery in severely affected patients.

SNfL levels were available in seven CIDP patients (median 106 pg/ml, range 5.7–162) and CSF NfL levels in three different patients (median 2506 pg/ml, range 1392–7527). Age-adjusted sNfL z-scores ranged from − 2.3 to + 3.6 (median + 1.6). All NfL measurements were obtained at the time of ICU admission before initiation of invasive procedures, to minimize confounding from ICU-related factors. Full details of laboratory, imaging, and electrophysiological data are provided in Table 4 and Supplementary Table S5.

**Table 4:** Diagnostic of patients with severe CAN at ICU

Variable	
Cerebral MRI, *n* (%) of *n* = 21	17 (81)
Normal or unspecific changes	13 (62)
Changes associated with CAN^a^	4 (19)
Spinal MRI, *n* (%) of *n* = 21	16 (76)
Normal or unspecific/age-related changes	10 (48)
Changes associated with CAN^b^	6 (29)
Plexus MRI, *n* (%) of *n* = 21	3 (14)
Normal or unspecific changes	0 (0)
Changes associated with CAN^c^	3 (14)
Laboratory values, *n* (%) of *n* = 21)	
Paraproteinemia in the urine	2 (10)
Paraproteinemia in the serum	6 (29)
Immunofixation in serum	21 (100)
Normal	16 (76)
Pathologic	5 (24)
ANA in serum elevated	2 (10)
Ganglioside antibodies, testing performed in serum	20 (95)
positive	3 (14)
Neurofascin antibodies, testing performed in serum	12 (57)
positive^d^	2 (10)
HbA1c, median (min/max, IQR) in %, *n* = 16	5 (5/8, 5–6)
HbA1c elevated^e^	5 (24)
NSE in serum, median (min/max, IQR) in µg/l, *n* = 3	21 (16/29, 16–29)
NfL in serum in pg/ml (min/max), *n* = 7	109 (5.7–162)
NfL in CSF in pg/ml, median (min/max), *n* = 3	2506 (1392–7527)
CSF analysis^f^, *n* (%) of *n* = 21	20 (95)
Cell count in cell/µl, median (min/max, IQR), *n* = 20	2 (0/9, 1–3)
Protein level in mg/l, median (min/max, IQR), *n* = 20	991 (320/5900, 643–1226)
Lactate level in mg/dl, median (min/max, IQR), *n* = 19	17 (11/35, 14–23)
Glucose in mg/l, median (min/max, IQR), *n* = 19	72 (31–137, 66–89)
Oligoclonal bands, *n* = 19	
No bands in CSF and serum	12 (57)
Oligoclonal IgG bands in CSF	0 (0)
OCB in CSF and serum with additional bands in CSF	0 (0)
Identical OCB in CSF and serum	7 (33)
Intrathecal Ig-synthesis	3 (14)

^a^Cranial nerve abnormalities

^b^thickening, and/or contrast enhancement of cauda equina fibers

^c^enlargement and/or increased signal intensity of nerve roots on T2-weighed MRI sequences and/or contrast enhancement of nerve roots

^d^One patient had NF155 and NF186 antibodies, one patient had NF155 antibodies

^e^HbA1c reference: < 6%

^f^One patient was diagnosed externally in 2005; there was no information available about CSF diagnostic

*ANA* antinuclear antibody, *ANCA* anti-neutrophil cytoplasmic antibody, *CAN* chronic immune mediated neuropathy, *CSF* cerebrospinal fluid, *IIFT* indirect immunofluorescence test, *NF*-*L* neurofilament light chain, *NSE* neuron-specific enolase, *OCB* oligoclonal bands

Histopathology was available from sural nerve biopsies in 13 of the 21 patients (CIDP = 11, MMN = 1, AN = 1). In the CIDP patients, sural nerve biopsy showed predominantly moderate-to-severe chronic axonal loss in 9/11 cases (4/11 moderate, 5/11 severe). CD8⁺ T-cell infiltration, indicative of ongoing immune pathology, was likewise moderate to severe in 7/11 patients (3/11 moderate, 4/11 severe). De- and remyelination markers, such as thinly myelinated fibers, were mostly mild (8/11, 73%), and acutely degenerating axons—showing ongoing nerve injury—were observed mildly in 8/11 (73%) and moderately in 1/11 (9%) of cases. These features did not vary consistently with acute (< 30 days), subacute (30–90 days), or chronic (> 90 days) biopsy timing after disease onset. One NF155/NF186-Ab positive patient showed moderate fiber loss, mild T-cell infiltration, and limited signs of de- and remyelination. One patient with MMN underwent sural nerve biopsy because CIDP was initially suspected at the time of diagnostic workup; the biopsy showed predominant signs of de- and remyelination. Complement deposition (C5b-9) on endoneurial capillaries was observed in all patients’ sural nerves with available staining (11/13, CIDP = 9, MMN = 1, AN = 1), predominantly mild; 1 + (5/11) to moderate; 2 +  + (5/11) in extent, with one patient showing strong deposition. A detailed description of each patient is provided in Table 5. No relevant associations were observed between ENG score or clinical outcome (mRS, INCAT) and fiber density, whereas CD8⁺ T-cell infiltration in sural nerve biopsy showed moderate positive correlations with mRS (0.3 95% CI − 0.4;0.8, *n* = 12) and INCAT (0.3 95% CI − 0.5;0.8, *n* = 8) at last follow-up, as well as with ENG score (0.3 95% CI − 0.3; 0.9, *n* = 7) at last follow-up, suggesting a possible association between increased inflammatory infiltration, worse functional outcome, and greater axonal damage; however, sample sizes were too small, precluding firm conclusions (Supplementary Table S6).

**Table 5:** Histopathological findings of sural nerve biopsy in patients with CAN

Diagnosis	Diagnosis2	Loss of myelinated fibers	De- and remyelination	Acuity	T-cell infiltration	Complement	ICU admission to biopsy (days)	Symptom onset to biopsy (days)	Immunotherapy before biopsy	Additional features
Possible CIDP	CIDP spectrum	+ +	+	+	+ + +	n.a	0	acute (16)	–	
Possible CIDP	CIDP spectrum	+ +	+ +	–	+	+ +	90	chronic (104)	Steroids	
CIDP	CIDP spectrum	+ +	+ +	+	+ +	+ +	21	chronic (212)	IVIG, PLEX	
A-CIDP	CIDP spectrum	+ + +	+	+	+ + +	n.a	30	subacute (69)	Steroids. IVIG, PLEX AZA	
Possible CIDP	CIDP spectrum	+ + +	–	–	+ +	+	243	subacute (71)	IVIG	
A-CIAP	CIDP spectrum	+ + +	+	+	+ + +	+	33	subacute (65)	IVIG, RTX, CPM	
A-CIDP	CIDP spectrum	+ +	+	+	+	+ + +	17	Acute (21)	IVIG, PLEX	
CIDP	CIDP spectrum	+ + +	+	+	+ +	+ +	61	Chronic (1891)	Steroids, IVIG	
A-CIDP	CIDP spectrum	+	+	+ +	+	+	138	Chronic (323)	Steroids, IVIG	
A-CIDP	CIDP spectrum	+ + +	+	+	+ + +	+ +	66	Chronic (117)	Steroids, IVIG	
CIDP	CIDP spectrum	+	+	+	+	+	2	Subacute (35)	n.a	
NF155/NF186 AN	Nodopathy	+ +	+	-	+	+ +	16	Subacute (74)	IVIG, PLEX	Granular deposits in EM
MMN	Other	+	+ + +	+	+	+	7	chronic (3891)	–	Granular deposits in EM

Timing of biopsy was classified as acute (< 30 days), subacute (30–90 days), or chronic (> 90 days) from symptom onset

*CPM* cyclophosphamide, *EM* electron microscopy, *IVIG* intravenous immunoglobulin, *n*.*a*. not available, *PLEX* plasma exchange, *RTX* rituximab

### Immunotherapy of ICU-treated patients

**Fig. 1:**
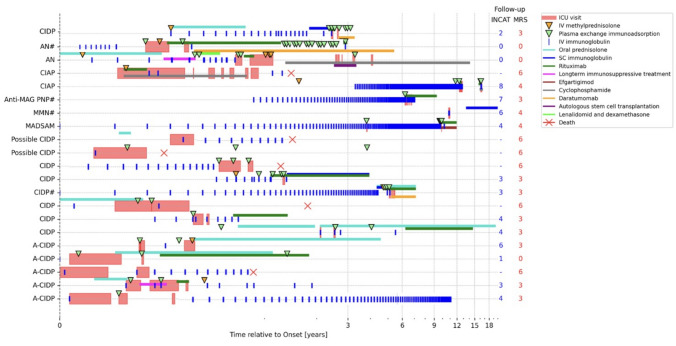
Swimmer plot illustrating all immunotherapies applied to each ICU-treated patient over the disease course, along with clinical outcomes at last follow up. *A*-*CIDP* acute-onset chronic inflammatory demyelinating polyneuropathy, *AN* autoimmune nodopathy, *Anti*-*MAG neuropathy* anti-myelin-associated glycoprotein neuropathy, *CIAP* chronic inflammatory axonal polyneuropathy, *ICU* intensive care unit, *INCAT* inflammatory neuropathy cause and treatment, *MADSAM* multifocal acquired demyelinating sensory and motor neuropathy, *MMN* multifocal motor neuropathy, *mRS* modified Rankin scale, *#* monoclonal gammopathy of undetermined significance

All ICU-treated patients received *first-line immunotherapy* within a median of 1 month (range: −129) from symptom onset including IVIG/SCIG (95%), steroids (62%), and/or PLEX (81%) (Supplementary Table 1 and Fig. 1). Prior to ICU admission, 67% (*n* = 14) of patients had already been treated, mainly with IVIG (19% *n* = 4) or IVIG in combination with steroids (33% *n* = 7). During ICU stay, most were treated with a combination therapy of IVIG and PLEX (57%), (Supplementary Table 1, and Fig. 1). The majority (62%, *n* = 13) required *“escalation”* therapy (rituximab: 62%, cyclophosphamide: 14%), initiated after a median of 8 months (range: 1.8–124.6) from first-line therapy. Of these, 24% remained treatment refractory and progressed within a shorter interval (median: 3 months, range: 1–21) to *intensified “escalation”* therapy (daratumumab: 14%, efgartigimod: 5%, autologous stem cell transplantation: 5%), including patients with MADSAM (*n* = 1), CIDP (*n* = 2) and AN (*n* = 2).

In the subgroup of patients with CIDP, 53% (*n* = 8) received “escalation” therapy (all rituximab); 20% (*n* = 3) required “intensified escalation” therapy (daratumumab 13%, efgartigimod 7%). Time from initiation of first-line therapy to “escalation” therapy was longer than in the overall cohort (median: 17 months; range: 2.5–115), while time to initiation of intensified “escalation” therapy (median: 3 months, range: 2–5) was comparable.

### Outcome of ICU-treated patients

**Fig. 2:**
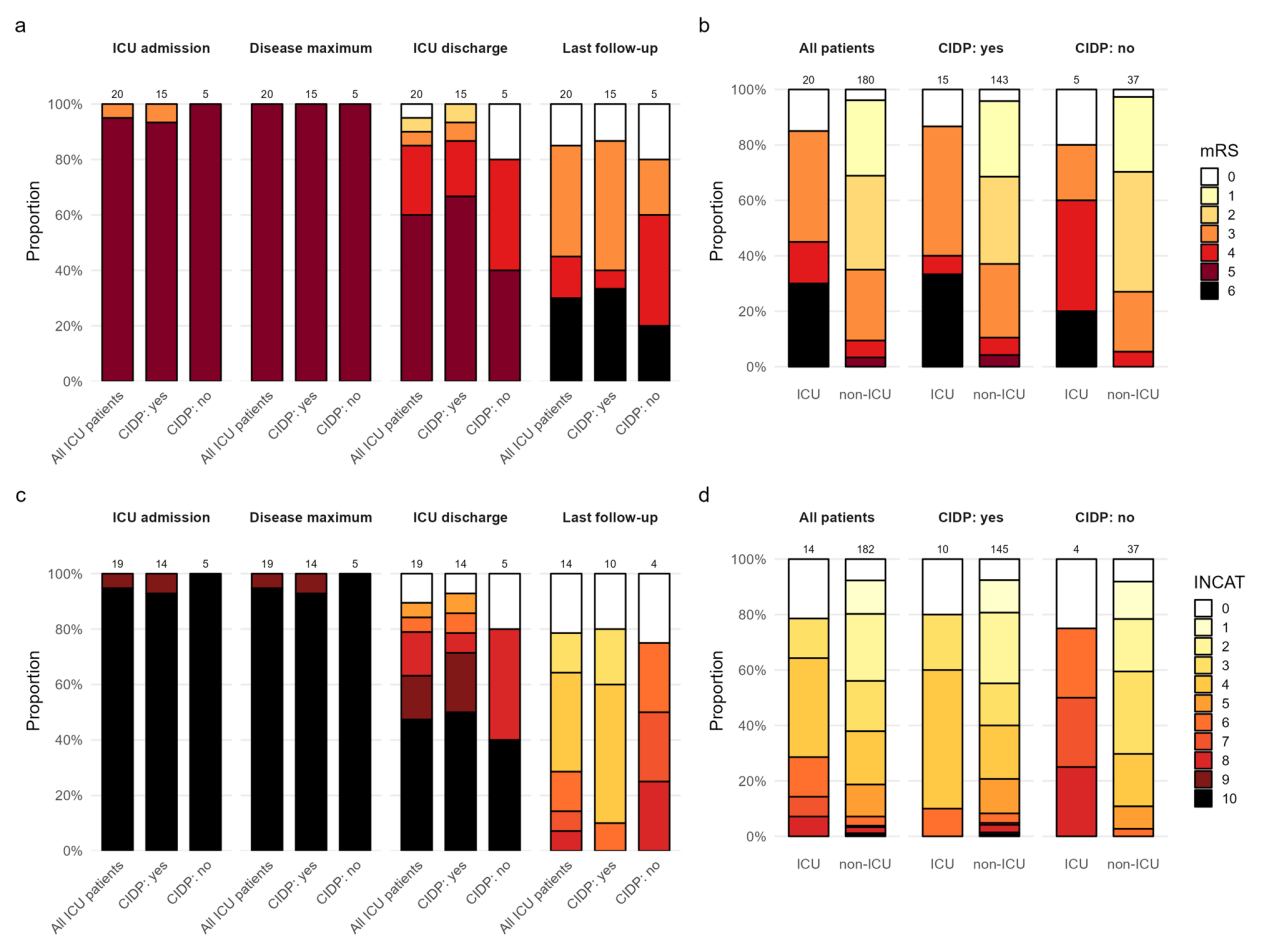
Stacked barplot of outcome data (mRS and INCAT), comparing matched ICU and non-ICU-treated patients. Longitudinal and cross-sectional distribution of functional outcomes using mRS and INCAT in age, sex, and diagnosis matched ICU-treated and non-ICU-treated CAN patients. **a** Distribution of mRS score among matched CAN ICU patients at ICU admission, disease maximum, ICU discharge, and last follow-up, shown for all ICU patients and stratified by CIDP subgroup. **b** Comparison of mRS scores between matched ICU and non-ICU CAN patients at last follow-up, overall and stratified by CIDP subgroup. **c** Distribution of INCAT disability score among matched CAN ICU patients at ICU admission, disease maximum, ICU discharge, and last follow-up, shown for all ICU patients and stratified by CIDP subgroup. **d** Comparison of INCAT score between matched ICU and non-ICU CAN patients at last follow-up, overall and stratified by CIDP subgroup. *AN* autoimmune nodopathy, *CAN* chronic autoimmune neuropathies, *INCAT score* inflammatory neuropathy cause and treatment disability score, *mRS* modified rankin scale

At ICU admission, the majority of patients (*n* = 18/21, 86%) had an mRS of 5 and an INCAT of 10. At disease nadir, the INCAT scores were the same, and one patient deteriorated in terms of mRS from 3 at ICU admission to 5. There was already partial recovery at ICU discharge compared to disease nadir, as 7/21 patients improved in terms of mRS, 9/21 in terms of INCAT. About one-third of patients (6/21, 29%) died within 2 years after ICU admission. At last follow-up, after a median of 19 months (range 1–157) since ICU discharge (Table 2), all of the other 15 patients further improved substantially in both outcome scores, with a median change of 2 units (95% bootstrap CI − 2;. − 5) in mRS and − 6 units (95% bootstrap CI − 8; − 5) in INCAT, compared to nadir (Fig. 2a and 2c). In survivors, the mRS at nadir was 5 for all patients and decreased to a median of 3 (range 0–4) at last follow-up, median INCAT from 10 for all patients to 4 (range 0–8). Notably, both patients with AN achieved the most favorable outcomes at last follow-up (mRS 0 and INCAT 0–1). ICU disease severity scores showed that scores generally increased from ICU admission to disease maximum and decreased toward ICU discharge, with broadly comparable patterns observed between subgroups (Supplementary Fig. S2).

Of 19 patients requiring initial invasive ventilation, 12 (63%) remained ventilator dependent at final ICU discharge. Among them, four were later decannulated; one patient remained tracheostomy ventilated after declining escalation therapy; and another continued NIV by choice. Six CAN patients (29%) still tracheotomized at ICU discharge had died by follow-up (median 216 days post-ICU, range: 22–554), with no available data on decannulation or cause of death (Table 2). Notably, only one of the six deceased patients (16%) received “escalation” therapy with rituximab.

Longer time from therapy to escalation was not consistently and not independently associated with worse short or long-term outcomes, based on binary logistic regression. Adjusting for potential confounders (sex, age, acute onset, tumor), delaying treatment by 12 months for each did not relevantly alter the odds of mRS > 2 at ICU discharge (OR 1.0, 0.91; 1.1) or at follow-up (OR 1.0, 0.9–1.1), or the odds for INCAT > 3 or death (OR 1.1, 1.0–1.2 at ICU discharge, 1.0, 0.9–1.1 at the last follow-up) (Supplementary Table S7).

Longer time from onset to ICU admission was not independently associated with worse long-term outcomes or death, based on logistic regression. Adjusting for sex and age at onset, each 1 year longer did not increase the odds of mRS > 2 (OR 1.0, 95% CI 1.0–1.1), and the odds of a one-category-higher mRS score (cOR 1.1, 95% CI 0.9–1.5), the odds of INCAT > 3 or death (OR 1.0, 95% CI 1.0–1.1), the odds of a one-category-higher INCAT score (cOR 1.1, 95% CI 0.9–1.4), or the odds of death alone (OR 1.0, 95% CI 0.9–1.0) (Supplementary Table S8).

## Comparison of ICU-treated and non-ICU-treated CAN patients

### Comparison of baseline characteristics

ICU-treated patients had shorter intervals from disease onset to study inclusion and from onset to initiation of first therapy. In addition, acute onset and a preceding infection as a suspected trigger were more frequent among ICU-treated patients, whereas prior treatment before ICU admission was less common in this group (Supplementary Table S1).

Propensity score matching resulted in a matched cohort of 20 ICU-treated and 186 non-ICU-treated patients (Table 1). In the matched cohort, standardized mean differences indicated persistent differences between ICU- and non-ICU-treated patients for acute onset, preceding infections, and age at onset, while time since study inclusion, time from onset to first treatment, and prior IVIG use were lower in ICU-treated patients (Table S1, Supplementary Fig. S4).

### Comparison of outcome

Although surviving ICU-treated patients showed substantial improvement from disease nadir, they exhibited higher mRS and INCAT scores at last follow-up than matched non-ICU patients across the overall cohort and within CIDP and non-CIDP subgroups (overall matched non-ICU: median mRS 2, range 0–5, median INCAT 3, range 0–8) (Fig. 2b and 2d). Adjusting for all variables with remaining differences after matching, ordinal logistic regression showed that ICU-treated patients still had worse functional outcome than non-ICU-treated patients—as shown by an increase in odds of a one-category-higher mRS (cOR 8.7, 95% CI 1.9–39.0) and higher INCAT, both when including death as the highest category (cOR 7.7, 95% CI 1.7–35.7) and when excluding death (cOR 8.2, 95% CI 1.0–71.0) Similar effect estimates were observed in adjusted binary logistic models. These results are not substantially impacted by varying follow-up intervals, as shown by adjustment for time from onset to last follow-up (Supplement Table S9). This is further illustrated in Supplement Fig. S5a/b, with the exception that patients who died did so within a relatively short time after ICU admission.

### Clinical characteristics, treatment, and outcome of literature-based ICU cohort

Based on a review of 27 studies, we identified 43 ICU-treated patients with CAN. Most (51%, *n* = 22) had typical CIDP (21% with acute onset), one MGUS-associated CIDP, 33% (*n* = 14) AN, one anti-MAG neuropathy, and 12% (*n* = 5) MMN. Like our cohort, most patients were male (65%) with a median age of 62 years; 82% required mechanical ventilation (vs. 95% in our ICU cohort). Immunotherapy was reported in 40 patients (93%): all received first-line therapy (77% IVIG, 67% steroids, 60% PLEX) mostly combined (31/40, 78%). Long-term immunosuppression was used in 18% (*n* = 8). Escalation therapies were administered in 23% (14% rituximab, cyclophosphamide: 9%), mainly in AN (16% vs. 7% in other CAN). One patient with AN received rituximab and bortezomib. Recovery occurred in 70% (*n* = 30, 35% full, 35% partial), while 4% (*n* = 2) had a poor outcome. Patients with AN tended to recover better (full recovery: 43% versus 31%, mortality rate: 21% versus 28%) (Supplementary Table S10).

Comparable to the findings in our ICU cohort, analyses of the literature-derived ICU cohort (available data in 29/43 patients) showed that a longer interval from symptom onset to ICU admission was consistently, but only modestly, associated with worse long-term outcomes at last follow-up. This association was observed in a binary model assessing the probability of death (OR 1.15, 95% CI 0.99–1.34) and in an ordinal model capturing shifts across outcome categories (death > poor outcome > partial recovery > full recovery; cOR 1.69, 95% CI 0.83–4.34; Supplementary Table S8). In both cohorts, full recovery more frequently occurred within short time intervals from symptom onset to ICU admission. In our cohort, deaths were likewise confined to this early time window, whereas in the literature-derived cohort, deaths were distributed across both early and later time intervals after disease onset (Supplement Table S7).

## Discussion

This retrospective study provides a comprehensive longitudinal analysis of patients with CAN requiring ICU treatment, focusing on therapeutic strategies and outcome measures compared to non-ICU patients. Most ICU patients with CAN did not respond sufficiently to first-line therapies and required “escalation” or intensified “escalation” therapies. About a third of patients died during follow-up, the other patients, despite prolonged and severe disease courses, showed substantially improved functional outcomes over time following intensified treatments. Nevertheless, long-term outcomes remain worse than in comparable non-ICU patients. Neuropathological assessment suggested variable cellular (e.g., CD8⁺) and humoral (e.g., C5b–9) components in our patients with CAN. These findings underscore the need for novel, mechanism-based therapeutic strategies, including combined first-line, “escalation”, and intensified “escalation” therapies in severely affected patients.

To date, only small case series [13] and isolated case reports [12, 38] have described CAN crises necessitating intensive care, and evidence on their frequency remains scare. While CAN-related ICU admissions were rare when expressed per patient-year, a relatively high annual ICU proportion was observed, possibly reflecting improved disease recognition and changes in treatment practices associated with expanding therapeutic options.

Despite their rarity, the high fatality rate underscores the clinical relevance and the need for specialized intensive care in dedicated centers. Within the ICU patients, 53.3% with CIDP and 28.5% with other CAN did not respond to first-line therapies. Overall, 24% were resistant to long-term immunosuppression and “escalation” with rituximab or cyclophosphamide. By contrast, prior studies reported refractoriness to first-line therapies in approximately 20–25% of patients with CIDP [7, 39], with 10–15% considered resistant to all currently available treatments [7], suggesting that our ICU cohort represents a distinct and particularly treatment-refractory subgroup.

All acute-onset patients with CAN and one patient with CIAP with subacute onset had a preceding infection within 2–6 weeks before symptom onset, and most required ICU admission at first manifestation of disease. This supports a role of infection-related immune activation in acute deterioration, resembling GBS, where up to two-thirds of patients report a preceding infection [40]. However, as pathogen identification was often lacking, causality in CAN remains uncertain and warrants further investigation.

All ICU-treated patients with CAN presented with tetraparesis at disease nadir, and 95% required invasive-mechanical ventilation. The severity likely reflects extensive axonal damage, potentially explaining the poor response to first-line therapy and aligning with previous reports in treatment-refractory CIDP [9]. Additional factors including acute or progressive onset, CNS involvement, and CIDP variants [39, 41–43], present in several of our CAN patients, may also have contributed.

In our cohort, sNfL z-scores at ICU admission showed a wide range, with elevated levels observed in most patients, indicating varying degrees of axonal involvement. This aligns with recent data showing that higher sNfL levels in CIDP are linked to more severe axonal damage and a greater need for intensive immunotherapy [44]. Three different CIDP patients had markedly elevated CSF NfL levels at ICU admission, together with electrophysiological findings of axonal injury, which may have contributed to their later requirement for escalation treatments. However, according to the bi-compartment model, serum and CSF NfL reflect partly distinct biological processes and may also be influenced by systemic factors, limiting the specificity of sNfL in critically ill patients [45]. Thus, sNfL levels should be interpreted cautiously in critically ill patients due to possible systemic influences, yet all determinations in our cohort were performed at ICU admission before invasive procedures, at least limiting early ICU-related confounding. As reliable biomarker to identify CAN patients at risk for severe or refractory courses remain limited [46, 47], future studies should investigate whether combined serum and CSF NfL assessment can improve risk stratification.

Histopathological findings revealed moderate-to-severe chronic axonal loss in 82% of patients with CIDP with moderate-to-strong CD8^+^ T-cell infiltration in 64%, indicating ongoing immune activity. C5b-9 complement deposition on endoneurial capillaries was present in all patients with available staining, mostly mild to moderate. Likewise, previous studies found deposition of complement factors C3 on the myelin sheath in a small sample of CIDP patients [48, 49]. Moreover, complement inhibition therapy has improved muscle weakness in an experimental rat model of CIDP. Given the small number of biopsies, varying disease durations, and the potential influence of prior immunotherapies and different treatment durations on cellular composition, axonal damage, and de-/regeneration and deposition of key complement factors, these findings must be interpreted cautiously. While fiber density was not associated with outcome, moderate correlations between CD8⁺ T-cell infiltration and both functional outcome and axonal damage at last follow-up suggest that the extent of inflammatory activity may influence clinical outcome and the degree of axonal injury. However, these analyses were exploratory and must be interpreted with caution due to the small sample size, as mentioned before. The results may highlight the heterogeneity of these neuropathies and point to distinct pathophysiological patterns in CAN. Given the success of complement-targeted therapies in other neuromuscular diseases [50, 51], further investigating complement pathways in critically ill patients with CAN could inform mechanism-based, targeted therapeutic strategies and improve patient care.

Literature data from ICU-treated CAN patients largely mirror our findings, with most patients being older men, high rates of mechanical ventilation, and widespread use of first-line immunotherapy. Despite less frequent use of escalation therapies compared with our cohort, clinical improvement at follow-up was observed in most CAN patients, particularly in those with AN.

Although the majority of surviving ICU-treated patients in our cohort showed marked clinical improvement over time, long-term outcomes remained less favorable than in a matched non-ICU cohort, even when adjusting for all differences between the groups. Outcomes were also less favorable when compared to other CIDP cohorts with long-term follow-up [42].

Our data suggest that delay in treatment escalation was not independently associated with worse outcomes, and that even severe axonal damage observed during ICU treatment did not uniformly predict poor long-term recovery. However, among the six patients who died early after ICU admission, only one had received escalation therapy, indicating that the absence of early treatment intensification may still be relevant in the most severe disease courses. In line with prior studies linking early and intensified immunotherapy to improved long-term outcomes and reduced axonal damage [47], our findings support that early therapeutic escalation should be considered even in severe presentations. Moreover, the lack of outcome differences across varying intervals from symptom onset to ICU admission suggests that disease severity at presentation, rather than disease duration alone, may be more relevant for prognosis in critically ill patients.

Rituximab and cyclophosphamide, key “escalation” options [47], were administered more frequently in our cohort than in the literature-based ICU cohort and the non-ICU cohort. While intensified “escalation” therapies were exceptional in the literature-based ICU cohort, another German study reported frequent “escalation” or intensified “escalation” immunotherapy including bortezomib or stem cell transplantation in therapy-refractory patients with CAN [7, 9]. Assuming long-lived plasma cells contribute to treatment-resistance to rituximab [9], 3 patients in our cohort received daratumumab: One patient with CIDP improved under long-term rituximab–daratumumab combination, another patient with CIDP required daratumumab after rituximab but still needed frequent IA for stability. A third patient with NF155/NF186 AN improved after switching to daratumumab following rituximab [37]. Although plasma exchange and rituximab are usually effective in AN[5], both of our patients with AN required intensified “escalation” therapies, including daratumumab or autologous stem cell transplantation [36, 37]. One patient with MADSAM stabilized only with a combination of rituximab and the FcRn inhibitor efgartigmod, enabling discontinuation of PLEX. FcRN inhibition [24], complement inhibitors [52] and early reports of anti-BCMA [53] or CD19-targeted CAR-T-cell therapies in relapsed CIDP and AN [54] show the potential of highly specific and treatments. However, CAR T-cell therapy is not a suitable treatment option in intensive care settings due to its side effect profile, such as cytokine release syndrome or immune effector cell-associated neurotoxicity syndrome.

Together, these findings illustrate the heterogeneity and treatment complexity of severe, therapy-refractory CAN, and emphasize the urgent need to better understand the underlying pathophysiological mechanisms to implement tailored immunotherapies.

This study has some limitations. As a retrospective, single-center analysis, it includes a heterogeneous group of patients with CANs. While this variability reflects real-world clinical practice, it reduces the comparability between individual cases. The long recruitment period further contributes to heterogeneity, as diagnostic standards and the availability of investigations evolved over time (e.g., paranodal Ab assays, advanced immunotherapies). As (para)nodal Abs were only recently identified, nine earlier ICU patients were not tested, possibly leading to underestimation of AN cases. The absence of standardized “escalation” therapies in refractory CAN resulted in individualized approaches and variable responses. Retrospective assessment of ICU outcomes may also reduce accuracy. The small sample size and subtype variability further limit statistical power and generalizability. Paired serum–CSF NfL measurements were not available in our cohort, preventing an evaluation of potential CSF–serum NfL relationships or the application of bi-compartment modeling [45]. Interpretation of CMAP and SNAP amplitudes is limited by the possibility of reversible conduction failure during acute deterioration. The dynamic changes observed in several patients (including resolution or evolution of partial conduction blocks) indicate that reduced amplitudes may not always represent permanent axonal degeneration.

## Conclusion

Taken together, CAN can present with acute crises at different disease stages, resembling acute inflammatory neuropathies but with a high and early fatality rate. Among survivors, escalation and intensified immunotherapies treatments were more frequently administered, and most patients demonstrated substantial clinical improvement. However, overall outcomes remained worse compared with non-ICU patients. These findings underscore the need for tailored treatment strategies and reliable biomarkers that both identify patients at risk for severe or therapy-refractory courses and clarify predominant pathophysiologic mechanisms to guide individualized, mechanism-based therapy.

## Supplementary Information

Below is the link to the electronic supplementary material.

Supplementary file1 (PDF 942 KB)

## Data Availability

The data that support the findings of this study are available on request from the corresponding author. The data are not publicly available because of information that could compromise the privacy of research participants.

## Abbreviations

Abs: Antibodies
A-CIDP: Acute-onset chronic inflammatory demyelinating polyneuropathy
AN: Autoimmune nodopathy
Anti-MAG neuropathy: Anti-myelin-associated glycoprotein neuropathy
CAN: Chronic autoimmune neuropathies
CIAP: Chronic inflammatory axonal polyneuropathy
CPM: Cyclophosphamide
DADS neuropathy: Distal acquired demyelinating symmetric neuropathy
ENG score: Electroneurography score
GBS: Guillain–Barré syndrome
IA: Immunoadsorption
ICU: Intensive care unit
INCAT: Inflammatory neuropathy cause and treatment
IVIG: Intravenous immunoglobulin
MADSAM: Multifocal acquired demyelinating sensory and motor neuropathy
MGUS IgG: Monoclonal gammopathy of undetermined significance immunoglobulin G
MMN: Multifocal motor neuropathy
mRS: Modified Rankin scale
NCS: Nerve conduction study
sNFL: Serum neurofilament light chain
PLEX: Plasma exchange
SCIG: Subcutaneous immunoglobulin
SLONM: Sporadic late-onset nemaline myopathy
