# Supplementary material for: Clinical course and prognosis of chronic autoimmune neuropathies requiring intensive care: a retrospective cohort study
Source: J Neurol. 2026 Feb 25;273(2):165. doi: 10.1007/s00415-026-13694-4 (PMC12935830; doi:10.1007/s00415-026-13694-4)
Supplement: Supplementary file 1 — Supplementary file1 (PDF 942 KB) [file 415_2026_13694_MOESM1_ESM.pdf]

Supplemental Material

To

Clinical Course and Prognosis of Chronic Autoimmune Neuropathies Requiring Intensive Care: A retrospective cohort study

Hannah Preßler[1,2,3#], Lisa Schwarz[1#], Simon Streit[4], Annette Aigner[5], Alicia Schleicher[1], Frauke Stascheit[1,3], Friederike A. Arlt[1,2,6], Viktoria Zinnow[2], Tatjana Khorassani[1], Harald Prüss[1,2], Wolfgang Böhmerle[1], Andreas Meisel[1,3,7], Werner Stenzel[4], Franziska Scheibe[1,3,8]

[1] Department of Neurology with Experimental Neurology, Charité-Universitätsmedizin Berlin, Corporate Member of Freie Universität Berlin, Humboldt-Universität Berlin, Berlin, Germany

[2] German Center for Neurodegenerative Diseases (DZNE) Berlin, Berlin, Germany

[3] Neuroscience Clinical Research Center, Charité-Universitätsmedizin Berlin, Corporate Member of Freie Universität Berlin, Humboldt-Universität Berlin, Berlin, Germany

[4] Department of Neuropathology, Charité-Universitätsmedizin Berlin, Corporate Member of Freie Universität Berlin, Humboldt-Universität Berlin, Berlin, Germany

[5] Institute of Biometry and Clinical Epidemiology, Charité-Universitätsmedizin Berlin, Corporate Member of Freie Universität Berlin, Humboldt-Universität Berlin, Berlin, Germany

[6] Department of Laboratory Medicine and Pathology, Mayo Clinic, Rochester, USA

[7] Center for Stroke Research Berlin, Charité-Universitätsmedizin Berlin, Corporate Member of Freie Universität Berlin, Humboldt-Universität Berlin, Berlin, Germany

[8] Department of Neurology, Asklepios Klinik Hamburg Harburg, Hamburg, Germany

[#] These authors contributed equally to this work.

Correspondence to:

Hannah Preßler, Department of Neurology and Experimental Neurology, Charité-Universitätsmedizin Berlin, Phone: 030 450 660 560, E-mail: hannah.pressler@charite.de

Supplementary Methods

Literature review

A comprehensive literature search was carried out in MEDLINE/PubMed, covering articles published between March 1987 and February 2025. The search utilized MeSH terms, free-text keywords, and related search terms, including (CIDP OR "Chronic Inflammatory Demyelinating Polyneuropathy" OR "Multifocal Motor Neuropathy" OR “MMN” OR "Anti-Myelin Associated Glycoprotein Polyneuropathy" OR "Anti-MAG Peripheral Neuropathy" OR "Chronic Inflammatory Axonal Polyneuropathy" OR “CIAP” OR "autoimmune nodopathy") AND ("Respiratory Insufficiency" OR "respiratory failure" OR "ventilatory support" OR "mechanical ventilation" OR "intensive care"). Further relevant publications were identified by reviewing the reference lists of the selected articles. Only studies published in English were considered. Articles were excluded if they did not focus on CAN/AN, lacked primary data, were unavailable in full text, failed to provide medical history, or included other confounding factors leading to ICU instead of neuropathy-related ICU treatment. Additionally, two cases were excluded as they had already been published as case reports[1,2] and were further described in this ICU cohort.

Fig. S1 PRISMA flow chart illustrating number of studies screened, included, and excluded

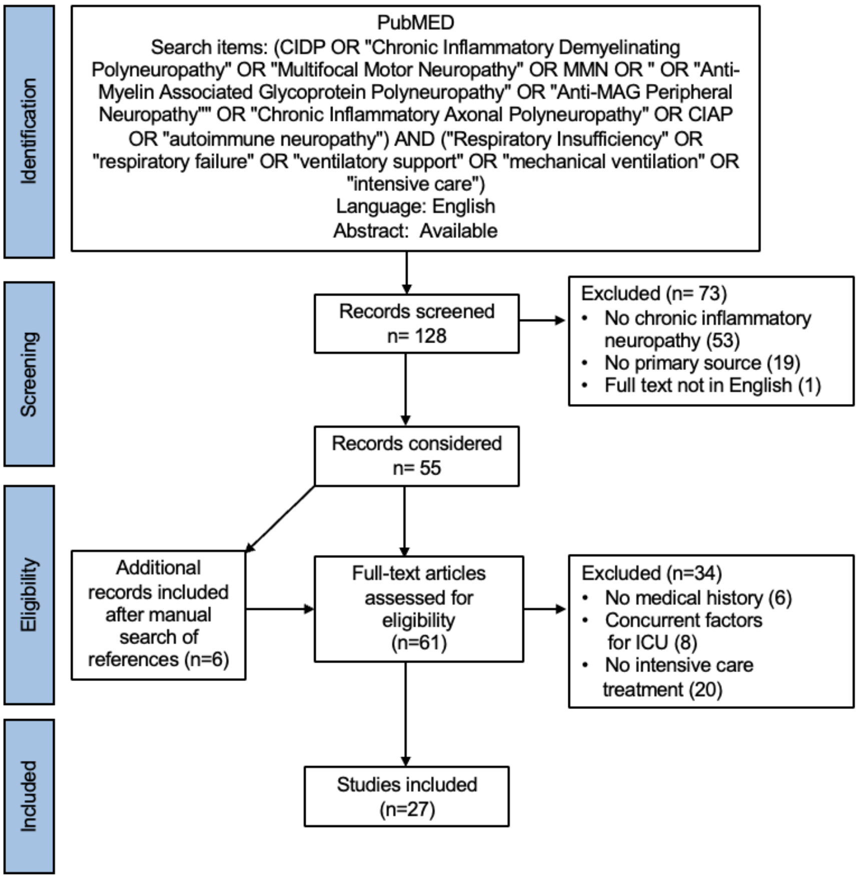

Supplementary Results

Fig. S2 ICU-scores (SOFA, APACHE II, TISS-28, SAPS II) of patients with CAN at ICU admission, disease maximum an ICU-discharge

Presentation of different ICU disease severity scores for all patients, patients with CAN without patients with CIDP, only patients with CIDP and patients with AN at different time points of ICU admission, disease nadir and ICU discharge. ~~Data are presented as median.~~ ~~Kruskal-Wallis test did not reveal statistical significance between the subgroups.~~

Abbreviations: AN = autoimmune nodopathy, APACHE II = Acute Physiology and Chronic Health Classification System II, CAN = chronic autoimmune neuropathies, CIDP = chronic inflammatory demyelinating polyneuropathy, ICU = intensive care unit, SAPS II = Simplified Acute Physiology Score II, SOFA = Sepsis-related Organ Failure Assessment, TISS-28 = Therapeutic Intervention Scoring System-28.

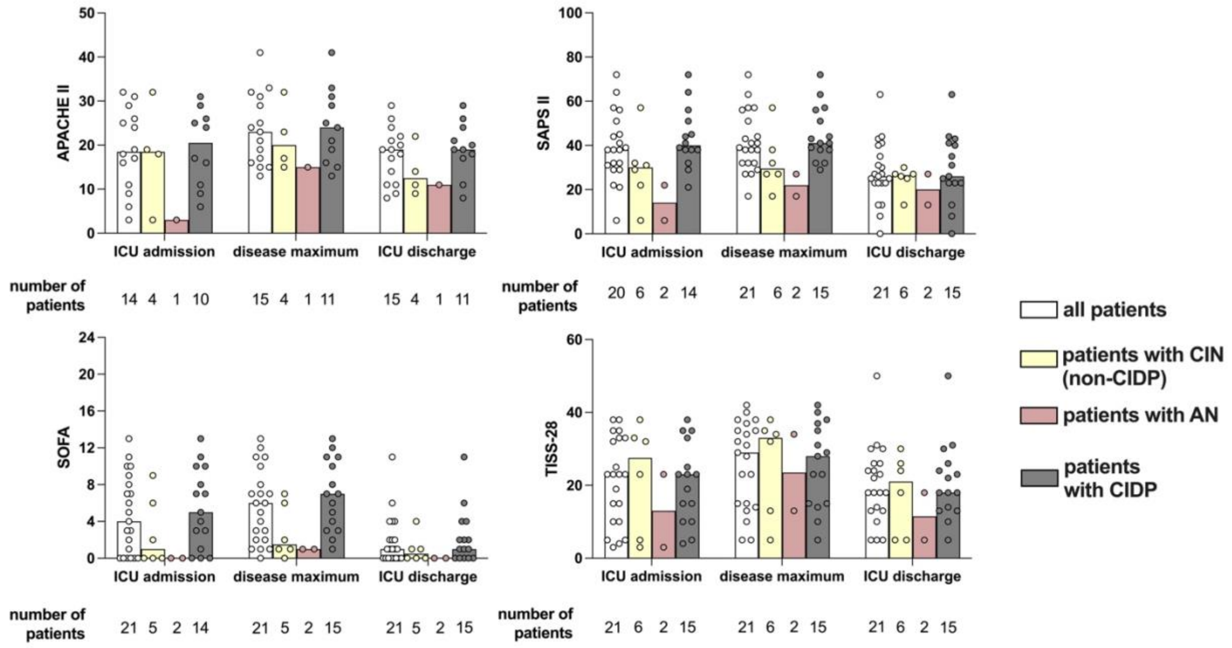

Fig. S3 Covariate balance before and after propensity score matching.

Shown are standardized mean differences of covariates before (unadjusted; red) and after propensity score matching (adjusted; blue). Covariates included age at study inclusion, sex, and diagnosis.

Propensity score matching substantially improved covariate balance across all included covariates, with post-matching SMDs close to zero and below the predefined threshold of 0.1 (dashed vertical line). Negative values indicate lower and positive values indicate higher values in ICU-treated patients.

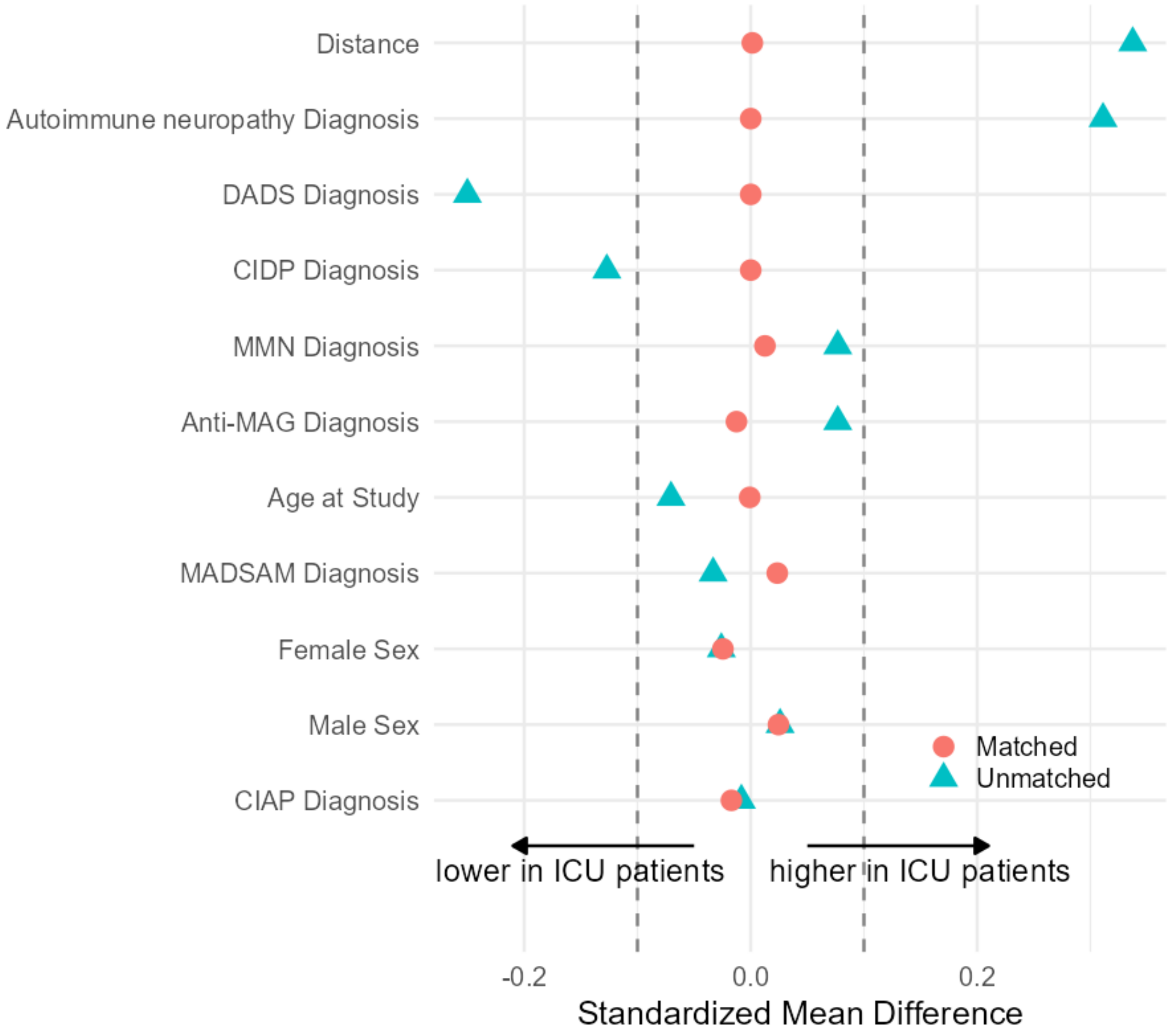

Fig. S4 Standardized mean differences (SMDs) of baseline characteristics between ICU- and non-ICU-treated patients after matching

The plot displays SMDs after propensity score matching for demographic variables, disease characteristics, and prior treatment. Negative values indicate lower and positive values indicate higher values in ICU-treated patients.

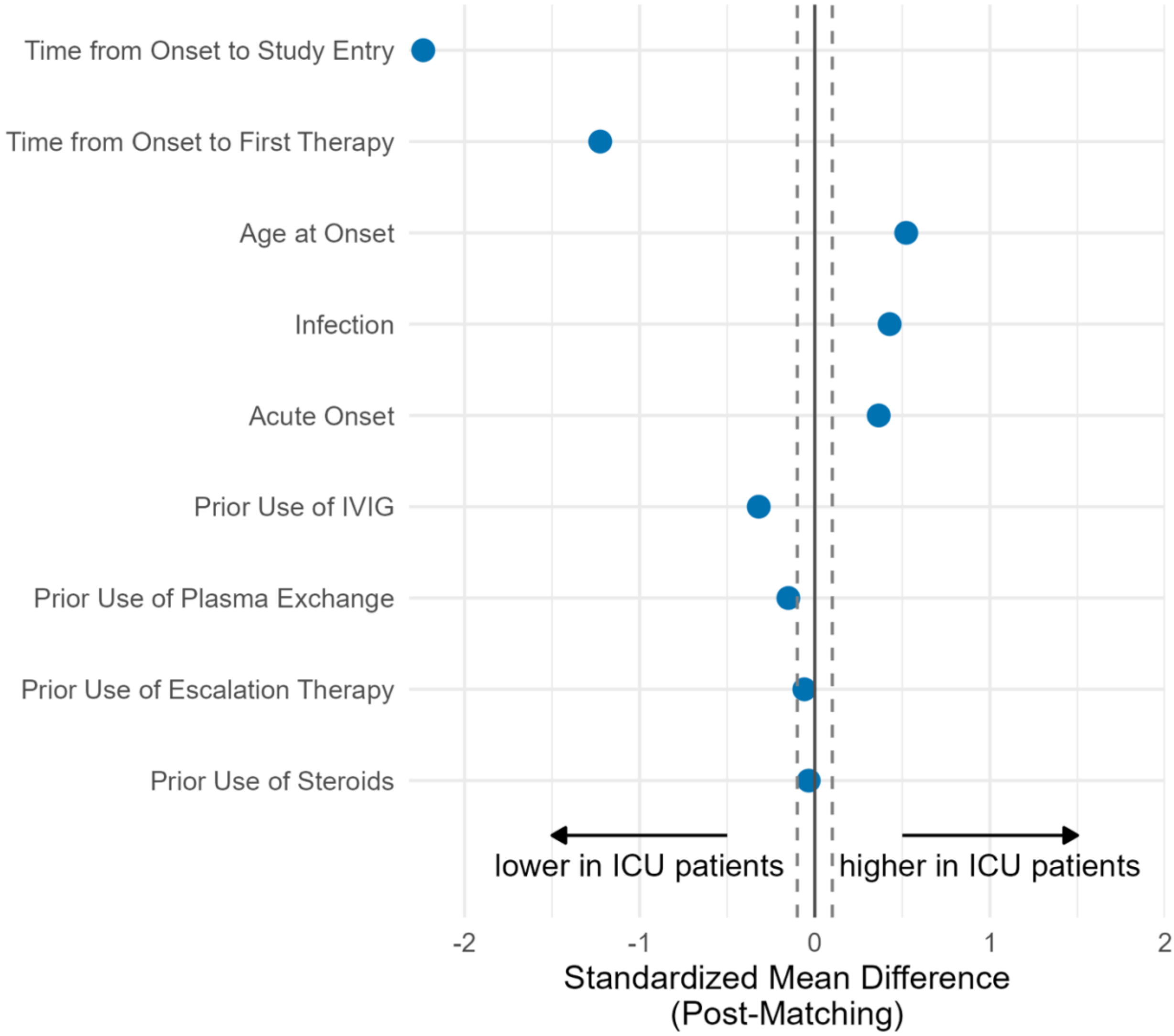

Fig. S5 (a). Modified Rankin Scale(mRS) over time from ICU-treated patients

Individual patient trajectories are shown, with mRS scores plotted over time since ICU admission (log-transformed scale). Circles indicate mRS at ICU admission, triangles at ICU discharge, and squares at last follow-up. Color represents diagnostic subgroups (anti-MAG neuropathy, autoimmune neuropathy, CIAP, CIDP, MADSAM and MMN).

Abbrevations: AN = autoimmune nodopathy; CIAP = chronic inflammatory axonal polyneuropathy, CIDP = chronic inflammatory demyelinating polyneuropathy, MADSAM = multifocal acquired demyelinating sensory and motor neuropathy, MAG = myelin associated glycoprotein, MMN = multifocal motor neuropathy.

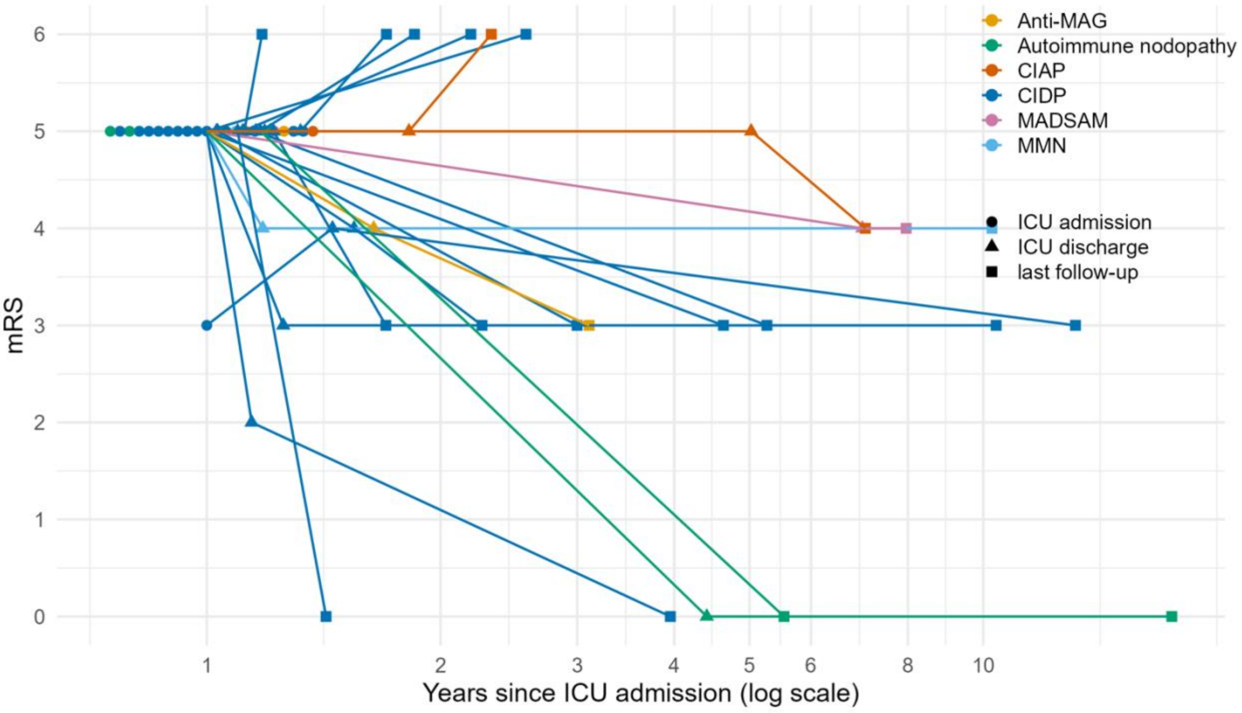

Fig. S5 (b) Modified Inflammatory Neuropathy Cause and Treatment disability (INCAT) over time from ICU-treated patients

Individual patient trajectories are shown, with INCAT scores plotted over time since ICU admission (log-transformed scale). Modification of INCAT score was done by including death as INCAT reaching from 0 (no symptoms) to 11 (death). Circles indicate INCAT at ICU admission, triangles at ICU discharge, and squares at last follow-up. Color represents diagnostic subgroups (anti-MAG neuropathy, autoimmune neuropathy, CIAP, CIDP, MADSAM and MMN).

Abbreviations: AN = Autoimmune nodopathy, CIAP = chronic inflammatory axonal polyneuropathy, CIDP = chronic inflammatory demyelinating polyneuropathy, MADSAM = multifocal acquired demyelinating sensory and motor neuropathy, MAG = myelin associated glycoprotein, MMN = multifocal motor neuropathy.

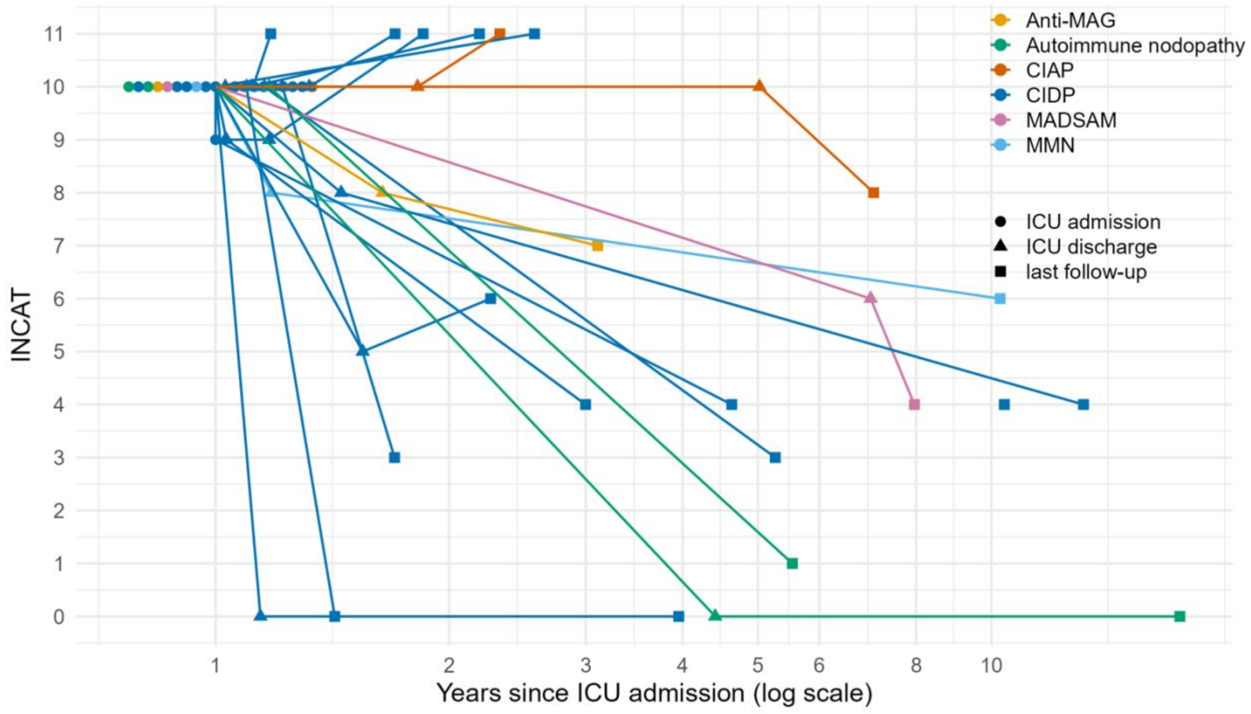

Table S1 Baseline characteristics and treatment trajectories of all ICU and Non-ICU patients with CAN

	Non-ICU cohort		ICU cohort		Total
	All patients	CIDP subgroup	All patients	CIDP subgroup[a]	
N°	256	200	21	15	277
**Age at symptom onset, median (Range)**<br>**Male sex, N (%)**	55 (14-87)<br>143 (56)	54 (14-87)<br>111 (56)	68 (27-83)<br>12 (57)	71 (27-83)<br>7 (48)	55 (2-87)<br>155 (56)
**Onset to therapy, months, Median [Range]**	22 (0-603.1)	20 (0-603.1)	1 (-6-129)	1 (0.0-37)	19 (5-603.1)
**Acute onset of symptoms (≤2months), N (%)**	22 (8.6)	19 (9.5)	10 (47.6)	8 (53.3)	32 (11.6)
**Suspected triggers, N (%)**					
None/ unknown	220 (85.9)	171 (85.5)	8 (38.1)	5 (33.3)	228 (82.3)
Infection	23 (9.0)	20 (10)	11 (52.4)	8 (53.5)	34 (12.3)
Vaccination	2 (0.8)	1 (0.5)	1 (4.8)	1 (6.7)	3 (1.1)
Medical procedures	11 (4.8)	8 (4)	1 (4.8)	1 (6.7)	12 (4.3)
**Treatment[b], N (%)**					
**First line therapy**					
Steroids	128 (50.6)	93 (46.5)	13 (61.9)	8 (53.3)	141 (51)
IVIG	224 (88.2)	179 (89.5)	20 (95.2)	14 (93.3)	244 (88.1)
PLEX/IA[c]	44 (17.3)	36 (18)	17 (81)	13 (87.6)	61 (22)
**First line therapy before ICU admission**					
Only steroids			2 (9.5)	2 (13.3)	2 (9,5)
Only IVIG			4 (19)	3 (20)	4 (19)
Steroids and IVIG			7 (33.3)	4 (26.7)	7 (33.3)
Steroids, IVIG and PLEX			1 (4.8)	0 (0)	1 (4.8)
**First line therapy in ICU setting**					
Only IVIG			3 (14.3)	2 (13.3)	3 (14.3)
Steroids + IVIG			2 (9.5)	(0)	2 (9.5)
Steroids + IVIG + PLEX			1 (4.8)	1 (6.7)	1 (4.8)
Steroids + PLEX			3 (14.3)	2 (13.3)	3 (14.3)
IVIG + PLEX			12 (57.1)	10 (66.7)	12 (57.1)
**"Escalation" therapy, N (%)**					
Long-term immunosuppressants	29 (11.3)	23 (11.5)	3 (14.3)	1 (6.7)	3 (1)
Rituximab	18 (7)	12 (6)	13 (61.9)	8 (53.3)	13 (4.7)
Cyclophosphamide	5 (2)	2 (1)	3 (14.3)	0 (0)	3 (1)
**"Intensified escalation" therapy, N (%)**					
Daratumumab	0 (0)	0 (0)	3 (14.3)	2 13.3)	3 (1)
Efgartigimod	0 (0)	0 (0)	1 (4.8)	1 (6.7)	1 (0.4)
Autologous stem cell transplantation	0 (0)	0 (0)	1 (4.8)	0 (0)	1 (0.4)
**Time from first line therapy to "escalation" therapy, in months, Median [Range]**			8.4 (1.8-124.6)	17.3 (4.1-60.24)	8.4 (1.8-124.6)
**Time from first line therapy to "intensified escalation" months, Median [Range]**			30.8 (29.4-62.7)	62.7 (30.8 – 117.2)	30.8 (29.4-62.7)

Data are presented as median [range] or frequency (%) as appropriate.

[a] including CIDP variants (MADSAM n=1) [b]multiple selection possible. [c]one patient needed plasma exchange for clinical stabilization every one to two weeks over several months.

Abbreviations: CAN = chronic autoimmune neuropathy; CIDP = chronic inflammatory demyelinating polyneuropathy; IA = immunoadsorption, IVIG = intravenous immunoglobulins, long-term IST = long-term immunosuppressants; PLEX = plasma exchange.

Table S2 Symptoms of patients with severe CAN at ICU admission, disease maximum, ICU discharge and last follow-up

Variable	n (%) of n=21
**Admission to ICU, % of n=21**	
Arm paresis	21 (100)
Leg paresis	20 (95)
Tetraparesis	20 (95)
asymmetric	4 (19)
symmetric	16 (76)
Muscle tonus, n=20	
normal	1 (5)
hypotonia	19 (90)
Reflexes, n=20	
hyporeflexia/areflexia arms	3 (14)
hyporeflexia/areflexia legs	3 (14)
hyporeflexia/areflexia arms and legs	14 (67)
Pathological reflexes	1 (5)
Sensory symptoms	13 (62)
Cranial nerve involvement[a,b]	8 (38)
oculomotor disorder	3 (14)
facial palsy	5 (24)
bulbar palsy	1 (5)
Dysarthria	10 (48)
Dysphagia	12 (57)
Dysphonia	6 (29)
**Disease maximum, n (%) of n=21**	
Tetraparesis	21 (100)
asymmetric	2 (10)
symmetric	19 (90)
Muscle tonus, n=21	
hypotonia	21 (100)
Reflexes, n=20	
hyporeflexia/areflexia arms	1 (5)
hyporeflexia/areflexia legs	4 (19)

hyporeflexia/areflexia arms and legs	15 (71)
Pathological reflexes	1 (5)
Sensory symptoms	10 (48)
Cranial nerve involvement[a,b]	10 (48)
oculomotor disorder	5 (24)
abducens nerve palsy	3 (14)
facial palsy	5 (24)
bulbar palsy	1 (5)
Dysarthria	10 (48)
Dysphagia	11 (52)
Dysphonia	7 (33)
**ICU discharge, n (%) of n=21**	
Arm paresis	18 (86)
Leg paresis	19 (90)
Tetraparesis	18 (86)
asymmetric	3 (14)
symmetric	15 (71)
Muscle tonus, n=20	
normal	5 (24)
hypotonia	15 (71)
Reflexes, n=19	
normal	2 (10)
hyporeflexia/areflexia arms	3 (14)
hyporeflexia/areflexia arms and legs	14 (67)
Pathological reflexes	1 (5)
Sensory symptoms	8 (38)
Cranial nerve involvement[b]	5 (24)
Bulbar palsy	1 (5)
facial palsy	3 (14)
Dysarthria	5 (24)
Dysphagia	8 (38)
Dysphonia	6 (29)

**Last follow-up, n (%), n=15**	
Leg paresis	11 (52)
Tetraparesis,	10 (48)
asymmetric	1 (5)
symmetric	9 (43)
Muscle tonus, n=14	
normal	12 (57)
hypotonia	2 (10)
Reflexes	
normal	6 (29)
hyporeflexia/areflexia arms	1 (5)
hyporeflexia/areflexia legs	1 (5)
hyporeflexia/areflexia arms and legs	7 (33)
Pathological reflexes	1 (5)
Sensory symptoms	7 (33)
Cranial nerve involvement	1 (5)
abducens nerve palsy	1 (5)
Dysarthria	0 (0)
Dysphagia	0 (0)
Dysphonia	0 (0)
Walking distance[c] at last follow-up, n (%), n=15	
not able to walk	2 (10)
<10 meters	2 (10)
<50 meters	3 (14)
100-200 meters	3 (14)
Up to 1000 meters	1 (5)
No walking limitations	4 (19)

[a]multiple selection possible; [b]one patient with Ig4-pseudotumor in the medulla oblongata presented with cranial nerve palsy after resection of the pseudotumor, rather suggesting a periprocedural complication than a manifestation of neuropathy; [c]walking distance with or without assistive devices (i.e. rollator).

Abbreviation: ICU = intensive care unit.

Table S3 Comorbidities of patients with severe CAN at ICU

Variable[a]	n (%) of n=21
Number of patients with comorbidities	20 (95)
Cardiovascular	10 (48)
Autoimmune	3 (14)
Neurological	6 (29)
Orthopedic	8 (38)
Psychiatric	2 (10)
Tumor disease[b]	5 (24)
colorectal cancer	3 (14)
breast cancer	1 (5)
lung cancer	1 (5)
urothelial carcinoma	1 (5)
Diabetes mellitus	5 (24)
Others[c]	9 (43)

[a]multiple selection possible. [b]one patient was diagnosed with colorectal and lung cancer. [c]others: hypothyroidism n=2, thalassemia n=1, Ig4-related pseudotumor n=1, MGUS=4, COPD=1, folic acid deficiency n=1, vitamin B12 deficiency n=2, atopic eczema n=1.

Abbreviations: COPD = chronic obstructive pulmonary disease, MGUS = Monoclonal gammopathy of undetermined significance.

Table S4 ICU complications of patients with severe CAN

Variable	n (%) of n=21
Infectious complications	
Pneumonia	18 (86)
Urinary tract infection	13 (62)
Colitis	2 (10)
Sepsis	8 (40)
Pneumothorax	1 (5)
Catheter associated thrombosis	7 (33)
Acute renal failure with necessity of dialysis	2 (10)
Renal insufficiency without necessity of dialysis	2 (10)
Hypotension	12 (57)
Cardiopulmonary resuscitation	6 (29)
Severe cardiac arrythmia	1 (5)
Embolic incidence	2 (10)
Severe bleeding	3 (14)
Surgical complications	5 (24)
CIP/CIM	3 (14)
Pleural effusions	1 (5)

Allergic reaction	3 (14)
Transfusion-dependent anemia	7 (33)
Severe electrolyte dearrangement	3 (14)
Others[a]	9 (43)

[a]priapism with suspected cavernosal thrombosis n=1, infectious ascites n=1, delirium n=2, toxic dermatitis n=1, hypernatremia due to diabetes insipidus centralis n=1, cardiogenic shock due to fulminant myocarditis n=1, drug-induced pancreatitis n=1, multiple catheter-associated thrombosis with consecutive severe venous condition n=1.

Abbreviations: CIM = critical-illness myopathy, CIP = critical-illness polyneuropathy.

Table S5 Electrophysiological data of patients with severe CAN requiring ICU treatment

		**Demyelinating parameters**	**Axonal parameters**							**EMG**	**ENG Score**	**Conduction block** [c]
	**Diagnosis**	**EAN/PNS criteria** [a]	**CMAP in mV (reduction in %)**				**SNAP in uv (reduction in %)**			**PSA (%)**[b]		
Nr			**Median**	**Ulnar**	**Tibialis**	**Peroneus**	**Median**	**Ulnar**	**Sural**			
**At intensive care unit**												
1	AN	definite	4.5 (35.7)	2.5 (64.3)	1.5 (50.3)	0 (100)	0 (100)	4 (60)	0 (100)	50	4	Partial CB ulnar nerve
2	AN	definite	6.4 (8.6)	8.1 (0)	12.7 (0)	4.9 (2)	1.3 (93.5)	2.6 (87)	21 (0)	70	0	-
3	CIDP	definite	0.1 (97.8)	0.1 (99)	0 (100)	0 (100)	3.5 (32.7)	0 (100)	0 (100)	80	4	-
4	A-CIAP	no	0.2 (96)	0.9 (85.9)	0 (100)	0.1 (99.5)	0 (100)	0 (100)	0 (100)	40	4	-
5	CIAP	no	0.9 (87.6)	1.9 (71.6)	0.6 (70)	0.4 (58)	2 (75)	9 (0)	1 (0)	20	4	Partial CB ulnar/median nerve
6	Anti-MAG	definite	0.1 (98)	0.2 (97.1)	0 (100)	0 (100)	4.4 (53.1)	2.9 (51.7)	1.8 (0)	20	n.a.	Partial CB ulnar/median nerve
7	MMN	definite	1.9 (72.6)	0.2 (97)	0 (100)	0.7 (72.8)	10.7 (0)	9.9 (1)	2.3 (23.3)	20	3	-
8	A-MADSAM	definite	0.6 (86)	0.8 (88.6)	0 (100)	0 (100)	0 (100)	0 (100)	0 (100)	0	4	Partial CB ulnar/median nerve
9	A-CIDP	possible	0.4 (94.4)	0.2 (97)	0 (100)	0 (100)	0 (100)	0.9 (83.8)	0 (100)	30	4	-
10	CIDP	not applicable	0 (100)	0 (100)	0 (100)	0 (100)	0 (100)	0 (100)	0 (100)	50	4	-
11	CIDP	definite	0.2 (97)	0.1 (98.6)	0.2 (90)	0 (100)	0 (100)	6.5 (0)	0 (100)	60	4	-
12	CIDP	definite	3.6 (48.6)	7.5 (0)	10.9 (0)	2.6 (35)	9.5 (52.5)	13.6 (32)	3.9 (35.0)	n.a.	3	-
13	CIDP	definite	1.3 (73.2)	2 (72.6)	0 (100)	0 (100)	0 (100)	0 (100)	0 (100)	20	4	-
14	CIDP	definite	0.1 (99)	0.5 (92.9)	0 (100)	0 (100)	6.8 (15)	7.8 (0)	3.2 (0)	40	3	-
15	CIDP	definite	6.6 (0)	2.2 (71.1)	1.9 (69.1)	0.9 (54.8)	0 (100)	1.1 (87.2)	0.4 (82.6)	40	4	Partial CB ulnar nerve
16	A-CIDP	definite	0.7 (90.6)	1.1 (84)	0 (100)	0 (100)	0 (100)	0 (100)	0 (100)	50	4	-
17	A-CIDP	definite	5.8 (17.1)	4 (42.9)	0.1 (98.1)	2 (50)	6.5 (67.5)	11.4 (43)	4.6 (23.3)	20	3	-
18	CIDP	definite	0.3 (96.1)	0.9 (87.6)	0 (100)	0 (100)	0 (100)	1.6 (84.4)	0 (100)	30	4	-
19	A-CIDP	not applicable	0 (100)	0 (100)	0 (100)	0 (100)	0 (100)	0 (100)	0 (100)	50	4	-
20	A-CIDP	definite	3.2 (54.3)	5.9 (15.7)	3.5 (14.3)	2.3 (0)	0 (100)	15.2 (0)	0 (100)	0	4	-
In total			**0.7 (89)**	**0.9 (86)**	**0 (100)**	**0 (100)**	**0 (100)**	**2.1 (84)**	**0.2 (100)**	**40**	**4**	
**At follow-up**												

1	AN	definite	11.2 (0)	9.5 (0)	1.5 (50.3)	0.4 (76.2)	9.6 (0)	9.5 (0)	0 (100)	0	1	-
2	AN	definite	6.4 (8.6)	12.8 (0)	12.7 (0)	4.2 (16.0)	9.4 (53)	15.8 (21)	21 (0)	n.a.	0	-
3	CIDP	definite	1.1 (75.4)	3.9 (46.6)	0.9 (75.9)	0.2 (84.4)	3.5 (32.6)	0 (100)	0 (100)	30	4	-
5	CIAP	no	1.9 (60)	71.6 (86)	1.2 (70.1)	0 (100)	6.1 (0)	4.4 (0)	0 (100)	30	4	-
6	MMN	definite	5.4 (22.9)	0 (100)	0.6 (78)	0.1 (90)	5.4 (32.5)	22.8 (0)	3.7 (0)	10	3	-
12	CIDP	definite	10.3 (0)	10 (0)	7.6 (0)	1.8 (55)	11.9 (40.5)	23 (0)	7 (0)	n.a.	1	-
13	CIDP	definite	9.1 (0)	5.6 (23.3)	0 (100)	0.1 (91.2)	0 (100)	0 (100)	0 (100)	0	3	-
15	CIDP	definite	6.6 (0)	2.2 (71.1)	1.9 (69)	0.9 (54.8)	0 (100)	1.1 (87.2)	0.4 (82.6)	40	4	CB ulnar nerve
16	A-CIDP	definite	9.6 (0)	7.9 (0)	1.6 (71.9)	1.1 (26.5)	4.3 (52.8)	1 (90)	0 (100)	n.a.	3	Parital CB ulnar nerve
17	A-CIDP	definite	7.4 (0)	8.9 (0)	0.1 (98.1)	2.3 (0)	19.6 (0)	18.5 (0)	15.2 (0)	10	n.a.	-
20	A-CIDP	definite	8.8 (0)	8.4 (0)	6.5 (0)	2.7 (0)	7.3 (0)	0 (100)	0 (100)	n.a.	2	-
**In total**			**7.4 (0)**	**7.9 (0)**	**1.9 (69.1)**	**0.9 (55)**	**6.1 (40.5)**	**4.4 (21)**	**23.3 (20)**	**10**	**3**	

Electrophysiological data are presented for each patient. Signs of demyelination was assessed according to EAN/PNS criteria. Axonal damage was evaluated based on CMAP and SNAP amplitudes, their relative reductions, and the ENG score. Acute nerve damage was determined by the presence of pathological spontaneous activity. Abbreviations: A-CIDP = acute-onset chronic inflammatory demyelinating polyneuropathy, A-MADSAM = acute multifocal acquired demyelinating sensory and motor neuropathy, AN = autoimmune nodopathy, Anti-MAG = anti-myein associated glycoprotein neuropathy, CIAP = chronic inflammatory axonal polyneuropathy, CIDP = chronic inflammatory demyelinating polyneuropathy, CB = conduction block, CMAP = compound muscle action potential, ENG = electroneurography, EMG = electromyography, MMN = multifocal motor neuropathy, SNAP = sensory nerve action potential, PSA = pathological spontaneous activity.

a. In two patients with severe sensorimotor axonal-demyelinating polyneuropathy, assessment according to EAN/PNS criteria was not possible.

b. Pathological spontaneous activity (PSA) was defined as fibrillations and positive sharp waves in more than 10% of needle sites. PSA severity was graded as follows: mild (PSA in 1–3 of 10 muscles, i.e. 10–30 %), moderate (4–6 muscles, 40–60 %), and marked (7–10 muscles, 70–100 %).

c. According to the electrophysiological criteria of (partial) nerve condution block[3]

Table S6 Spearman’s rank correlation between histopathological findings and clinical outcome and between histopathological findings and degree of axonal damage (ENG score)

	mRS score	INCAT score	ENG score at ICU	ENG score at last follow-up
Fiber density				
Spearman correlation coefficient	0.1	0.0	0.1	-0.1
95% CI	-0.5; 0.6	-0.8; 0.8	-0.6; 0.8	-1.0; 1.0
N	12	7	11	6
$CD8^+$ T-cell infiltration				
Spearman correlation coefficient	0.3	0.3	0.2	0.3
95% CI	-0.4; 0.8	-0.5 ;0.8	-0.5; -0.7	-0.3 ; 0.9
N	12	8	11	7

Spearman’s rank correlation coefficients along with a 95% bootstrap confidence interval (CI) to quantify the association between histopathological findings (degree of nerve damage and quantification of inflammatory infiltration) and clinical outcome (mRS and INCAT score) at last follow-up, and between histological findings and axonal damage (ENG score) assessed at disease maximum at ICU and last follow-up.

Table S7 Effect of 12-months' time difference from therapy start to "escalation" therapy on short- and long-term outcome.

Outcome	Model	Unadjusted OR (95% CI)	Adjusted OR (95% CI)	Unadjusted OR (95% CI)	Adjusted OR (95% CI)
		**Shortterm (at ICU discharge)**	**Shortterm (at ICU discharge)**	**Longterm (at last FU)**	**Longterm (at last FU)**
mRS > 2	Binary logistic	1.0 (1.0; 1.1)	1.0 (0.9; 1.1)	1.1 (1.0; 1.1)	1.0 (0.9; 1.1)
mRS	Ordinal logistic	0.9 (0.7; 1.2)	Model cannot be fitted	1.5 (1.1; 2.4)	1.3 (0.7; 2.3)
INCAT > 3 or death	Binary logistic	1.0 (1.0; 1-1)	1.0 (0.9; 1.1)	1.1 (0.9; 1.7)	1.1 (1.0; 1.2)
INCAT with death highest category	Ordinal logistic	0.9 (0.7; 1.2)	Model cannot be fitted	1.4 (1.1; 2.1)	Model cannot be fitted
INCAT (excluding death)	Ordinal logistic	0.9 (0.7; 1.2)	Model cannot be fitted	2.0 (1.3; 3.9)	Model cannot be fitted

Displayed are unadjusted and adjusted odds ratio (OR) estimates along with 95% confidence intervals (CI) illustrating the effect per 12-months difference from therapy initiation to "escalation" treatment on short-term outcomes at ICU discharge and long-term outcomes at last follow-up.

Functional outcomes were assessed using the modified Rankin Scale (mRS) and the Inflammatory Neuropathy Cause and Treatment (INCAT) disability score. Binary logistic regression was applied for dichotomized outcomes (mRS > 2; INCAT > 3 or death) and ordinal logistic regression was used for ordinal outcomes (mRS; INAT, with death coded as the highest category or excluded, as specified). Adjusted models were controlled for: age at study inclusion, acute disease onset, presence of tumor, and sex. Models that could not be fitted due to insufficient data or model assumptions are indicated accordingly. OR estimates > 1 indicate a higher odds of worse functional outcome.

Table S8 Effect of time from disease onset to ICU admission on clinical outcomes.

**Data Source**	**Outcome**	**Model**			**Unadjusted OR (95% CI)**	**Adjusted OR (95% CI)**
			**Time from onset to ICU < 12m**	**Time from onset to ICU >= 12m**		
ICU cohort	mRS > 2	Binary logistic	8 (66.7%)	9 (100.0%)	1.0 (1.0; 1.1)	1.0 (1.0; 1.01)
	mRS	Ordinal logistic			1.1 (0.9; 1.30	1.1 (0.90; 1.5)
	INCAT > 3 or death	Binary logistic			1.0 (1.0; 1.1)	1.0 (1.0; 1.1)
	INCAT with death highest category	Ordinal logistic			1.1 (0.9; 1.3)	1.1 (0.9; 1.4)
	INCAT (excluding death)	Ordinal logistic			2.1 (1.4; 4.1)	2.1 (1.4; 3.9)
	death	Binary logistic	5 (41.7%)	1 (11.1%)	1.0 (0.9; 1.0)	1.0 (0.9; 1.0)
Literature-derived cohort	death	Binary logistic	4 (19.0%)	4 (50.0%)	1.2 (1.00; 1.35)	1.1 (1.0; 1.3)
	Death or poor outcome > partially recovered/Improved > recovered/full remission	Ordinal logistic			1.7 (0.9; 4.2)	1.7 (0.8; 4.3)

Displayed are unadjusted and adjusted odds ratio (OR) estimates along with 95% confidence intervals (CI) illustrating the effect of time from disease onset to ICU admission, analyzed per 12 months . Short- and long-term outcomes were assessed using the modified Rankin Scale (mRS) and the Inflammatory Neuropathy Cause and Treatment (INCAT) disability score. Binary logistic regression was applied for dichotomized outcomes (mRS > 2; INCAT > 2 or death; death) and ordinal logistic regression was used for ordinal outcomes (mRS; INCAT with death coded as the highest category, INCAT excluding death; and ordinal clinical outcome categories (death or poor outcome, partially recovered/improved, recovered/full remission) in the literature derived cohort. Adjusted models were controlled for age at disease onset and sex. Results are presented separately for the ICU cohort and the literature-derived cohort. Models that could not be fitted or where data were insufficient are not reported. OR estimates > 1 indicate a higher odds of worse functional outcome.

Table S9 Comparison of functional outcome at last follow-up of matched ICU- and non-ICU treated patients

Outcome	Model	OR unadjusted (95% CI)	OR adjusted [1] (95% CI)	OR adjusted[2] (95% CI)
mRS at last FU > 2	Binary logistic	11.4 (3.2; 41.2)	12.5 (3.21; 48.7)	30.8 (7.0; 135.6.)
mRS at last FU	Ordinal logistic	7.1 (1.9;; 26.8)	7.2 (1.93; 26.7)	8.7 (1.9; 39)
INCAT at last FU > 3 or death	Binary logistic	5.3 (1.8; 15.4)	6.6 (2.1; 20.8)	9.3 (2.2; 39.1)
INCAT at last FU with death highest category	Ordinal logistic	4.9 (1.6; 15.3)	5.2 (1.7; 15.6)	7.7 (1.7; 35.7)
INCAT at last FU (excluding death)	Ordinal logistic	2.2 (0.7; 7.53)	2.9 (0.8; 9.9)	8.2 (1.0; 71.0)

Displayed are unadjusted and adjusted odds ratio (OR) estimates along with 95% confidence intervals (CI) for poor functional outcome at last follow-up. Outcomes were assessed using the modified Rankin Scale (mRS) and the Inflammatory Neuropathy Cause and Treatment (INCAT) disability score. Binary logistic regression was applied for dichotomized outcomes (mRS > 2; INCAT > 2 or death) and ordinal regression was used for ordinal outcomes (mRS; INCAT with death coded as the highest category; INCAT excluding death).

[1] models adjusted for time from disease onset to last follow-up

[2]models adjusted for time from disease onset to study inclusion, time from disease onset to first therapy, acute disease onset, age at disease onset, and exposure to specific therapies including intravenous immunoglobulins (IVIG), plasmapheresis, escalation therapy, steroids, and prior infections before ICU admission. .

OR estimates > 1 indicate a higher odds of worse functional outcome.

Table S10 Literature review of reported cases of patients with severe CAN with intensive care treatment

Ref. ‡	Diagnosis	Age/ Gender	Treatment	Onset to ICU (m)	Invasive mechanical ventilation	Course	Relevant comorbidities	Follow-Up	Outcome
[4]	CIDP	62, F	Steroids	3	yes	Progressive		1,5 mo	Partially recovered
[4]	CIDP	66, M	IVIG, Steroids, Azathioprine	6	yes	Progressive			Died (pulmo- infection)
[5]	CIDP	79, F	IVIG, Steroids, Azathioprine	15	yes*	Relapsing		n.d.	Recovered
[5]	A-CIDP	57, F	IVIG	n.d.		n.d.		n.d.	Recovered
[5]	CIDP	71, M	PLEX, IVIG, Cyclophosphamide	5	yes*	Relapsing		8 mo	Recovered, relapse 8 m later
[5]	CIDP	68, M	Steroids	10	yes*	Progressive		n.d.	Recoverd
[6]	CIDP	49, M	IVIG	60	no	Relapsing	Pulmonary hypertension	n.d.	Died (respiratory failure)
[7]	A-CIDP	27, F	IVIG, PLEX, Steroids	2,5	yes	Progressive	SLE	14 yrs	Recovered
[8]	A-CIDP	51, F	PLEX, Steroids, Mycophenolat mofetil	n.d.	yes	Relapsing		2 yrs	Improved
[8]	A-CIDP	38, F	PLEX, IVIG, Steroids	12	yes	Relapsing		4 yrs	Improved
[8]	CIDP	69, M	IVIG, Steroids	12	yes	Progressive	Congestive heart failure, emphysema	2 yrs	Died (myocard infarction)
[8]	CIDP	66, M	PLEX, IVIG, Steroids	n.d.	yes	Progressive	Essential thrombocythemia	2 yrs	Partially recovered
[8]	CIDP	82, F	PLEX, Steroids	n.d.	yes	Relapsing	Pulmonary embolism	18 mo	Poor outcome
[8]	A-CIDP	66, M	PLEX, IVIG	n.d.	yes	Relapsing	SIRS	26 yrs	Partially recovered
[8]	A-CIDP	38, M	PLEX, IVIG, Steroids	n.d.	yes	Relapsing		13 yrs	Recovered
[9]	CIDP	60, M	PLEX, Steroids	36	yes	Progressive		6 mo	Partially recovered
[10]	A-CIDP	14, M	IVIG, Steroids	15	yes*	Progressive		n.d.	Recovered
[11]	A-CIDP	42, M	IVIG, Steroids	4	yes*	Progressive	B-cell lymphoma, DM, ischemic heart disease, history of unexplained lower limb paraplegia	n.d.	Recovered
[12]	CIDP	53, F	IVIG, PLEX, Steroids Cylosporine	3	yes	Monophasic	DM	8 yrs	Recovered
[13]	A-CIDP	56, M	IVIG, Rituximab	0,5	yes*	Relapsing	n.d.	1 mo	Recovered

14	CIDP	54, M	IVIG, Steroids	1	yes	Progressive	SIADH	~ 2 mo	Partially recovered
15	A-CIDP/MGUS	71, F	IVIG, PLEX, Steroids, Methotrexate, Melphalan	<1	yes*	Progressive		18 mo	Partially recovered
16	CIDP	78, F	Steroids, IVIG, PLEX,	3	yes	Progressive			Died (sudden cardiac arrest)
17	Anti-MAG	76, M	n.d.	24	no	Progressive	Coronary artery disease	n.d.	Died (declined invasive ventilatory support)
18	MMN	72, M	IVIG, PLEX, Steroids (at the beginning)	1	yes	Relapsing	Adenocarcinoma lung	n.d.	Died pulmonary embolism
19	MMN	56, F	IVIG	4	yes	n.d.	Psoriasis, Hypertension, uterus tumor	n.d.	Partially recovered
20	MMN	62, M	IVIG, PLEX, Steroids (at the beginning), Cyclophosphamide	3	no	Progressive		n.d.	Transient mild improvement, poor outcome
21	MMN	64, M	IVIG	2	no	Relapsing	Campylobacter infection		Died (septic shock)
22	MMN	46, M	IVIG, PLEX, Steroids	18	yes	Progressive	Amyloidosis		Died (heart failure)
23	AN (NF140/186)	70's, M	IVIG, PLEX, Steroids	2	yes*	Monophasic	Low grade IgA lambda myeloma	9 mo	Recovered
24	AN (NF140/186)	61, M	IVIG, PLEX, Steroids, Rituximab, Cyclophosphamide	5	yes	n.d.			Recovered
24	AN (NF140/ 186)	50, F	PLEX, Steroids	1,8	yes	n.d.	Glomerulosclerosis		Partially recovered
25	AN (NF155/186)	71, M	n.d.	n.d.	yes	n.d.	Cervial myelopathy		Died (sepsis)
25	AN (NF155/186)	63, M	n.d.	n.d.	yes	n.d.	Cervial myelopathy, bullous pemphigoid		Partially recovered
26	AN (Pan-NF)	52, M	IVIG, PLEX, Steroids, Rituximab, Bortezomib	1,5	yes*	Progressive		3 mo	Partially recovered
	AN (Pan-NF)	83, M	IVIG, PLEX	n.d.	No	n.d.			Death after cardiac resuscitation and palliative care concept
27	AN (Pan-NF)	73, M	IVIG, PLEX, Steroids, Rituximab	1-2	yes	n.d.		23	Remission
27	AN (Pan-NF)	72, F	IVIG, PLEX, Steroids, Rituximab	2-3	yes	n.d.		9	Partially recovered
27	AN (Pan-NF)	46, M	IVIG, PLEX, Steroids	<1	no	n.d.		11	Complete remission
27	AN (Pan-NF)	74, F	PLEX	<1	No	n.d.		n.d.	Complete remission

28	AN (Pan-NF)	60's, F	IVIG, PLEX	<1	yes	Progressive	Nephrotic syndrome, SAB	n.d.	Death after therapy withdrawal
28	AN (Pan-NF)	70's, M	IVIG, PLEX, Steroids, Rituximab	<1	yes	Monophasic		9 months	Complete remission
29	AN (Pan-NF)	50, M	IVIG, PLEX, Cyclophosphamide	1.5	yes*	Progressive		10	Partially recovered

Abbreviations: Anti-MAG = Anti-Myelin-associated Glycoprotein neuropathy; AN = autoimmune neuropathy; (A-) CIDP = (Acute-onset) chronic inflammatory demyelinating polyneuropathy; DM = diabetes mellitus; m(o) = months; MGUS = Monoclonal Gammopathy of Undetermined Significance; IVIG = intravenous immunoglobulin; MMN = multifocal motor neuropathy; n.d. = no data; NF = Neurofascin; PLEX, plasma exchange; SAB = subarachnoid hemorrhage; SIADH = Syndrome Of Inappropriate Antidiuretic Hormone Secretion; SIRS = Systemic Inflammatory Response Syndrome; SLE = systemic lupus erythematodes; yrs = years. *Patient had a temporary tracheostomy a) In some patients more than one ventilatory event happened.

Note: Three patients did not receive invasive mechanical ventilation; two received non-invasive ventilation (one declined invasive ventilation), and one received only supplementary oxygen.

‡ **References:**

1. Scheibe F, Ostendorf L, Prüss H, et al. Daratumumab for treatment-refractory antibody-mediated diseases in neurology. *Eur J Neurol*. Jun 2022;29(6):1847-1854. doi:10.1111/ene.15266

2. Scheibe F, Alexander T, Pruss H, et al. Devastating humoral CIDP variant remitted by autologous stem cell transplantation. *Eur J Neurol*. Mar 2016;23(3):e12-4. doi:10.1111/ene.12896

3. American Association of Electrodiagnostic M, Olney RK. Guidelines in electrodiagnostic medicine. Consensus criteria for the diagnosis of partial conduction block. *Muscle Nerve Suppl*. 1999;8:S225-9.

4. Stojkovic T, De Seze J, Hurtevent JF, Fourrier F, Vermersch P. Phrenic nerve palsy as a feature of chronic inflammatory demyelinating polyradiculoneuropathy. *Muscle Nerve*. Apr 2003;27(4):497-9. doi:10.1002/mus.10361

5. Henderson RD, Sandroni P, Wijdicks EF. Chronic inflammatory demyelinating polyneuropathy and respiratory failure. *J Neurol*. Oct 2005;252(10):1235-7. doi:10.1007/s00415-005-0848-2

6. Kimber TE, Orrell RW, King RH, Ginsberg L. Pathological findings in a patient with ventilatory failure and chronic inflammatory demyelinating polyneuropathy. *J Peripher Nerv Syst*. Mar 2003;8(1):13-6. doi:10.1046/j.1529-8027.2003.03003.x

7. Hantson P, Kevers L, Fabien N, Van Den Bergh P. Acute-onset chronic inflammatory demyelinating polyneuropathy with cranial nerve involvement, dysautonomia, respiratory failure, and autoantibodies. *Muscle Nerve*. Mar 2010;41(3):423-6. doi:10.1002/mus.21543

8. Zivković SA, Peltier AC, Iacob T, Lacomis D. Chronic inflammatory demyelinating polyneuropathy and ventilatory failure: report of seven new cases and review of the literature. *Acta Neurol Scand*. Jul 2011;124(1):59-63. doi:10.1111/j.1600-0404.2010.01431.x

9. Tataroglu C, Ozkul A, Sair A. Chronic inflammatory demyelinating polyneuropathy and respiratory failure due to phrenic nerve involvement. *J Clin Neuromuscul Dis*. Sep 2010;12(1):42-6. doi:10.1097/CND.0b013e3181d80bdc

10. Jha S, Ansari M, Sonkar K, Paliwal V. Unusual features in chronic inflammatory demyelinating polyneuropathy: Good outcome after prolonged ventilatory support. *J Neurosci Rural Pract*. Jul 2011;2(2):171-3. doi:10.4103/0976-3147.83586

11. Haji K, Butler E, Royse C. A case of chronic inflammatory demyelinating polyneuropathy with reversible alternating diaphragmatic paralysis: case study. *Crit Ultrasound J*. Dec 2015;7(1):16. doi:10.1186/s13089-015-0033-5

12. Seino Y, Nakamura T, Hirohata M, Kawarabayashi T, Okushima T, Shoji M. Severe Chronic Inflammatory Demyelinating Polyneuropathy Ameliorated following High-dose (3 g/kg) Intravenous Immunoglobulin Therapy. *Intern Med*. Mar 15 2019;58(6):855-859. doi:10.2169/internalmedicine.1723-18

13. Christani M. [Respiratory failure can be seen in chronic inflammatory demyelinating polyneuropathy: a case report]. *Lakartidningen*. Feb 3 2022;119Andningssvikt hos patienter med CIDP kräver skyndsam diagnos.

14. Taguchi Y, Takashima S, Sasahara E, et al. Inappropriate secretion of antidiuretic hormone in a patient with chronic inflammatory demyelinating polyneuropathy. *Intern Med*. Jan 2005;44(1):65-7. doi:10.2169/internalmedicine.44.65

15. Hayashi S, Nagamine S, Makioka K, Kusunoki S, Okamoto K. [A case of severe chronic inflammatory demyelinating polyradiculoneuropathy with monoclonal gammopathy of undetermined significance with alternating immunoglobulin class to IgM from IgA]. *Rinsho Shinkeigaku*. Sep 29 2016;56(9):593-9. doi:10.5692/clinicalneurol.cn-000889

16. Krasenbrink I, Kaps M, Blaes F. IVIg-induced acute polyneuroradiculitis in a patient with CIDP? *Eur J Neurol*. May 2007;14(5):e9. doi:10.1111/j.1468-1331.2007.01722.x

17. Tanariyakul M, Takaoka K, Takahashi T, Estaris J, Sumida K. Uncommon Presentation of IgM Monoclonal Gammopathy of Undetermined Significance (MGUS) and Anti-Myelin-Associated Glycoprotein (MAG)-Associated Demyelinating Peripheral Neuropathy as Respiratory Failure: A Case Report. *Cureus*. Jun 2024;16(6):e62865. doi:10.7759/cureus.62865

18. Beydoun SR, Copeland D. Bilateral phrenic neuropathy as a presenting feature of multifocal motor neuropathy with conduction block. *Muscle Nerve*. Apr 2000;23(4):556-9. doi:10.1002/(sici)1097-4598(200004)23:4<556::aid-mus15>3.0.co;2-g

19. Boonyapisit K, Katirji B. Multifocal motor neuropathy presenting with respiratory failure. *Muscle Nerve*. Dec 2000;23(12):1887-90. doi:10.1002/1097-4598(200012)23:12<1887::aid-mus16>3.0.co;2-y

20. Cavaletti G, Zincone A, Marzorati L, Frattola L, Molteni F, Navalesi P. Rapidly progressive multifocal motor neuropathy with phrenic nerve paralysis: effect of nocturnal assisted ventilation. *J Neurol*. Sep 1998;245(9):613-6. doi:10.1007/s004150050255

21. Galassi G, Albertini G, Valzania F, Barbieri A. Cranial nerve involvement as presenting sign of multifocal motor neuropathy. *J Clin Neurosci*. Dec 2012;19(12):1733-5. doi:10.1016/j.jocn.2011.12.030

22. Beydoun SR, Rison RA, Commins D. Secondary amyloidosis as a life-ending event in multifocal motor neuropathy. *Muscle Nerve*. Oct 2001;24(10):1396-402. doi:10.1002/mus.1162

23. Vallat JM, Mathis S, Magy L, et al. Subacute nodopathy with conduction blocks and anti-neurofascin 140/186 antibodies: an ultrastructural study. *Brain*. Jul 1 2018;141(7):e56. doi:10.1093/brain/awy134

24. Delmont E, Manso C, Querol L, et al. Autoantibodies to nodal isoforms of neurofascin in chronic inflammatory demyelinating polyneuropathy. *Brain*. Jul 1 2017;140(7):1851-1858. doi:10.1093/brain/awx124

25. Stengel H, Vural A, Brunder AM, et al. Anti-pan-neurofascin IgG3 as a marker of fulminant autoimmune neuropathy. *Neurol Neuroimmunol Neuroinflamm*. Sep 2019;6(5)doi:10.1212/nxi.0000000000000603

26. Fels M, Fisse AL, Schwake C, et al. Report of a fulminant anti-pan-neurofascin-associated neuropathy responsive to rituximab and bortezomib. *J Peripher Nerv Syst*. Dec 2021;26(4):475-480. doi:10.1111/jns.12465

27. Appeltshauser L, Junghof H, Messinger J, et al. Anti-pan-neurofascin antibodies induce subclass-related complement activation and nodo-paranodal damage. *Brain*. May 2 2023;146(5):1932-1949. doi:10.1093/brain/awac418

28. Fehmi J, Davies AJ, Walters J, et al. IgG(1) pan-neurofascin antibodies identify a severe yet treatable neuropathy with a high mortality. *J Neurol Neurosurg Psychiatry*. Oct 2021;92(10):1089-1095. doi:10.1136/jnnp-2021-326343

29. Burnor E, Yang L, Zhou H, et al. Neurofascin antibodies in autoimmune, genetic, and idiopathic neuropathies. *Neurology*. Jan 2 2018;90(1):e31-e38. doi:10.1212/wnl.0000000000004773
